# NPY_1_R-targeted peptide-mediated delivery of a dual PPARα/γ agonist to adipocytes enhances adipogenesis and prevents diabetes progression

**DOI:** 10.1016/j.molmet.2019.11.009

**Published:** 2019-11-16

**Authors:** Stefanie Wittrisch, Nora Klöting, Karin Mörl, Rima Chakaroun, Matthias Blüher, Annette G. Beck-Sickinger

**Affiliations:** 1 Universität Leipzig, Institute of Biochemistry, Brüderstraße 34, 04103 Leipzig, Germany; 2 Helmholtz Institute for Metabolic, Obesity, and Vascular Research (HI-MAG) of the Helmholtz Zentrum München at the University of Leipzig and University Hospital Leipzig, Ph.-Rosenthal-Str. 27, 04103 Leipzig, Germany; 3 Department of Medicine, University of Leipzig, Liebigstraße 20, 04103 Leipzig, Germany

**Keywords:** Adipocyte targeting, NPY_1_R, Peptide drug conjugate, Tesaglitazar, Type 2 diabetes, T2D, type 2 diabetes, PPARα/γ, peroxisome proliferator-activated receptor alpha/gamma, GPCR, G protein-coupled receptor, NPY_1_R, neuropeptide Y_1_ receptor, NPY, neuropeptide Y, tesa, tesaglitazar, tesa-NPY, [K^4^(GFLG-tesa), F^7^, P^34^]-NPY, Fabp4, fatty acid binding protein 4, FFA, free fatty acids, Cidec, cell death-inducing DEFA-like effector c, Mcp-1, monocyte chemotactic protein

## Abstract

**Objective:**

PPARα/γ dual agonists have been in clinical development for the treatment of metabolic diseases including type 2 diabetes and dyslipidemia. However, severe adverse side effects led to complications in clinical trials. As most of the beneficial effects rely on the compound activity in adipocytes, the selective targeting of this cell type is a cutting-edge strategy to develop safe anti-diabetic drugs. The goal of this study was to strengthen the adipocyte-specific uptake of the PPARα/γ agonist tesaglitazar via NPY_1_R-mediated internalization.

**Methods:**

NPY_1_R-preferring peptide tesaglitazar-[F^7^, P^34^]-NPY (tesa-NPY) was synthesized by a combination of automated SPPS and manual couplings. Following molecular and functional analyses for proof of concept, cell culture experiments were conducted to monitor the effects on adipogenesis. Mice treated with peptide drug conjugates or vehicle either by gavage or intraperitoneal injection were characterized phenotypically and metabolically. Histological analysis and transcriptional profiling of the adipose tissue were performed.

**Results:**

In vitro studies revealed that the tesaglitazar-[F^7^, P^34^]-NPY conjugate selectively activates PPARγ in NPY_1_R-expressing cells and enhances adipocyte differentiation and adiponectin expression in adipocyte precursor cells. In vivo studies using *db/db* mice demonstrated that the anti-diabetic activity of the peptide conjugate is as efficient as that of systemically administered tesaglitazar. Additionally, tesa-NPY induces adipocyte differentiation in vivo.

**Conclusions:**

The use of the tesaglitazar-[F7, P34]-NPY conjugate is a promising strategy to apply the beneficial PPARα/γ effects in adipocytes while potentially omitting adverse effects in other tissues.

## Highlights

• Tesaglitazar-NPY targets adipocytes via NPY_1_R receptor-mediated internalization.
• Peptide-drug conjugate is specifically delivered to NPY_1_R-expressing cells.
• Release of tesaglitazar in adipocytes activates PPARγ.
• Drug delivery enhances adipocyte differentiation and adiponectin expression.
• Peptide conjugate exhibits antidiabetic activity in vivo.

## Graphical abstract

**Figure:**
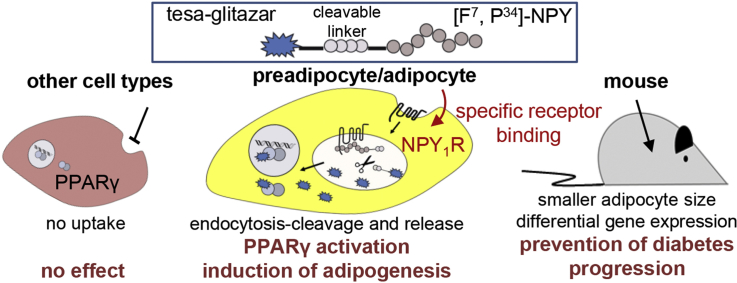

## 1 Introduction

Obesity is a global epidemic that continues to rise and consequently results in the increased occurrence of associated metabolic disorders such as type 2 diabetes (T2D) and cardiovascular diseases [[1], [2], [3]]. An imbalance between energy expenditure and energy intake leads to an increased storage of lipids in adipose tissue. This excess fat can either be stored in newly differentiated adipocytes, resulting in an increased cell number (hyperplasia), or in already existing adipocytes, causing enlarged cells (hypertrophy) [4]. While hyperplastic adipocytes seem to be metabolically risk-free, hypertrophy has been linked to the development of metabolic diseases [5,6]. The amount of adipocytes is mainly determined during childhood and adolescence, and as a result, hypertrophy is the preferred mechanism for the extension of adipose tissue as the ability of de novo adipogenesis is often exceeded in obese patients [4,7]. Peroxisome proliferator-activated receptor gamma (PPARγ) agonists are known for their outstanding anti-diabetic potential as they promote adipogenesis and lead to the development of small, metabolically healthy adipocytes [[8], [9], [10], [11], [12]]. However, these molecules further regulate a variety of processes in other cell types [13,14]. Therefore, the clinical application of some PPARγ agonists is accompanied by side effects including congestive heart failure, increased cardiovascular risk, cancer, or weight gain, which lead to complications in clinical trials and restrictions on their use (https://www.fda.gov/drugs/drug-safety-and-availability/fda-drug-safety-communication-avandia-rosiglitazone-labels-now-contain-updated-information-about and https://www.fda.gov/drugs/drug-safety-and-availability/fda-drug-safety-communication-updated-drug-labels-pioglitazone-containing-medicines) [[15], [16], [17], [18], [19]]. Experiments with lipodystrophic mice demonstrated that organs other than adipose tissue mainly contribute to these side effects [20]. Therefore, the selective targeting of PPARγ agonists to adipocytes represents a promising strategy for the development of safer insulin-sensitizing drugs and is needed to further use these agents in clinics.

Peptide ligands of G protein-coupled receptors (GPCR) are promising targeting moieties for selective cell delivery as they bind with high selectivity and affinity to their receptors, can easily be modified by chemical synthesis, lack antigenicity, and induce an efficient internalization in the target cell [[21], [22], [23]]. Peptide drug conjugates consist of three parts: a carrier peptide that specifically binds to the GPCR expressed on the targeted tissue, a cleavable linker that possesses extracellular stability and is cleaved after translocation to an intracellular compartment, and the drug molecule [24,25]. After activation of the GPCR, the peptide-receptor complex undergoes internalization, which is crucial for delivering the drug inside the cell. The internalized complex is then transferred to the endosome where the linker can be cleaved [26]. This leads to a free drug that can act on either a metabolic or transcriptional level to modulate cell behavior and activity. High levels of neuropeptide Y_1_ receptor (NPY_1_R) mRNA and protein were detected in human adipose tissue, 3T3-L1 preadipocytes, and adipocytes [[27], [28], [29]]. This expression was higher in obese patients [30]. In addition to adipose tissue, NPY_1_R can be found in vascular smooth muscle cells and the CNS, including the thalamus, hippocampus, and cerebral cortex [[31], [32], [33]]. Peptide ligand NPY (neuropeptide Y) can cross the blood–brain barrier in rats. However, it is unclear whether this is also the case in humans, as different studies demonstrated that the permeability is low [[34], [35], [36]]. Thus, NPY_1_R is a promising target for selective targeting of adipocytes. NPY stimulation of adipocytes leads to a mitogenic effect and enhanced cell proliferation [37,38]. In addition, a high dose of NPY promotes adipocyte differentiation via enhanced PPARγ expression, which might lead to additional beneficial effects and a combinatorial therapeutic approach of the peptide carrier and drugs [39].

Thiazolidinediones are the most well-known and frequently used class of PPARγ agonists, yet these molecules do not possess functional moieties suitable for attachment to peptides and therefore cannot be used in peptide drug conjugates [40]. The dual PPARα/γ agonist tesaglitazar (tesa), in contrast, contains a carboxyl function suitable for synthesis. This agonist is a promising anti-diabetic drug that was already shown to reduce insulin resistance in mice and humans [[41], [42], [43]]. The clinical investigation of tesa was discontinued in phase III because of safety concerns regarding renal dysfunction [16,44]. As these side effects were caused by the action of tesa on the kidney, a selective targeting of tesa to adipocytes could be a promising strategy to continue its clinical trials [45,46]. The advantage of dual PPARα/γ agonists is, that they, in addition to the insulin-sensitizing effects of PPARγ, improve lipid parameters and reduce cardiovascular complications associated with metabolic disorders through PPARα [16,47]. However, these effects are mainly mediated by PPARα expressed in the kidney, liver, muscle, and endothelial cells [16,45,48]. Therefore, we did not anticipate cardioprotective effects when tesa was selectively targeted to adipocytes and focused on PPARγ activity in the present study.

**Figure 1:**
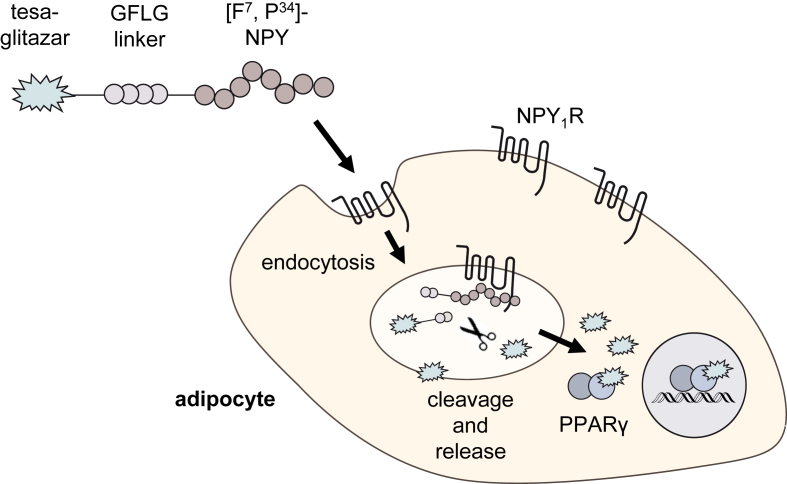
**Schematic illustration of adipocyte targeting by peptide drug conjugates.** NPY_1_R expressed on the adipocyte cell surface can potentially be used for the selective delivery of PPARγ agonists into adipocytes. The carrier peptide [F^7^, P^34^]-NPY is conjugated with a cleavable linker and tesa pharmacophore and can bind NPY_1_R, thereby triggering activation and subsequent internalization. The peptide-receptor complex undergoes endocytosis into the endosome, where the linker can be cleaved and tesa is released, which activates PPARγ and thus regulates transcription.

In this study, we aimed to transport tesa selectively into adipocytes by targeting it to NPY_1_R to develop a safe anti-diabetic drug that combines the beneficial effects of [F^7^, P^34^]-NPY and a dual PPARα/γ agonist while omitting adverse effects in other tissues (Figure 1). Therefore, we synthesized a peptide drug conjugate consisting of NPY_1_R-preferring ligand [F^7^, P^34^]-NPY, a cleavable GFLG linker, and tesa and tested this conjugate extensively in cell culture and *db/db* mice to determine its anti-diabetic potential.

## 2 Materials and methods

### 2.1 Materials

To synthesize peptides, N_α_-9-fluorenylmethoxycarbonyl (Fmoc)- and *tert*-butyloxycarbonyl (Boc)-protected amino acids were purchased from Orpegen (Heidelberg, Germany) and Iris Biotech (Marktredwitz, Germany). Rink amide resin, 1-hydroxybenzotriazole (HOBt), 2-cyano-2-(hydroxyimino) acetic acid ethyl ester (Oxyma), and N,N′-diisopropylcarbodiimide (DIC) were obtained from Iris Biotech. Thioanisole (TA) and ethanedithiol (EDT) were acquired from Fluka (Buchs, Switzerland). Acetonitrile (ACN), dichloromethane (DCM), and N,N′-dimethylformamide (DMF) were obtained from Biosolve (Valkenswaard, the Netherlands). Diethyl ether and ethanol were purchased from Scharlau (Barcelona, Spain). Hydrazine, piperidine, and trifluoroacetic acid (TFA) were purchased from Sigma Aldrich.

For cell cultures and cell culture-related assays, DMEM High Glucose and Ham's F12 cell culture media, Hanks' Balanced Salt Solution (HBSS), Dulbecco's Phosphate-Buffered Saline (DPBS), trypsin–EDTA, and an AdipoRed Adipogenesis Assay Kit were obtained from Lonza (Basel, Switzerland). Fetal calf serum (FCS) was purchased from Biochrom GmbH (Berlin, Germany). Penicillin, streptomycin, and hygromycin B were acquired from Invivogen (Toulouse, France). Biotin, pantothenate, dexamethasone (dex), triiodo-l-thyronine, human apo-transferrin, hydrocortisone, dimethyl sulfoxide (DMSO), probenecid, 4-(2-hydroxyethyl)-1-piperazineethanesulfonic acid buffer (HEPES), and Hoechst 33342 were obtained from Sigma–Aldrich (Taufkirchen, Germany). 3-isobutyl-1-methylxanthine (IBMX) was acquired from Fluka (St. Louis, MO, USA) and human insulin was obtained from Roche (Basel, Switzerland). Tesaglitazar, rosiglitazone, and tumor necrosis factor α (TNFα) were purchased from Biomol (Hamburg, Germany). Fluo2-AM and pluronic acid F-127 were acquired from TEFLabs (Austin, TX, USA). OptiMEM was obtained from Life Technologies (Basel, Switzerland). Lipofectamine 2000 and PowerSYBR Green were purchased from Thermo Fischer Scientific (Waltham, MA, USA). A Cignal PPARγ Reporter (luc) Kit, RNeasy Mini Kit, and QuantiNova Reverse Transcription Kit were obtained from Qiagen (Hilden, Germany) and the Dual-Luciferase System Kit from Promega (Madison, WI, USA). Six-well plates and 96-well plates were purchased from TPP AG (Trasadingen, Switzerland), and 8-well μ-slides were obtained from ibidi GmbH (Planegg, Germany).

### 2.2 Solid phase peptide synthesis (SPPS)

All the peptides were synthesized via a combination of automated solid-phase peptide synthesis (SPPS) with a Syro I peptide synthesizer (MultiSynTech) and manual couplings using the orthogonal Fmoc/tBu strategy on Rink amide resin (15 μmol scale, loading 0,7 mmol/g). Unmodified NPY (1), [F^7^, P^34^]-NPY (2), and Boc-[K^4^(Dde), F^7^, P^34^]-NPY were obtained by robot-assisted synthesis. Coupling reactions were then carried out twice with 8 eq *N*_*α*_-protected amino acids, in situ activated with equimolar amounts of Oxyma (2 min pre-incubation on resin) and DIC in DMF for 30 min. *Tert*-butyl (*t* Bu for Tyr, Ser, Asp, Glu, and Thr), trityl (Trt for Asn, Gln, and His), 2,2,4,6,7-pentamethyldihydrobenzofuran-5-sulfonyl (Pbf for Arg), and 4,4-dimethyl-2,6-dioxocyclohex-1-ylidenethyl (Dde for Lys) protection groups were used to protect the reactive side chains of the indicated amino acids. Deprotection of Fmoc was performed automatically with 40% (v/v) piperidine in DMF for 3 min and 20% (v/v) piperidine in DMF for 10 min.

After automated synthesis, deprotection of K^4^(Dde) (Boc-[K^4^(Dde), F^7^, P^34^]-NPY) was conducted 12 times using 2% (v/v) hydrazine in DMF for 10 min. Manual elongation of the *N*_*ε*_-group of K^4^ with the amino acids GFLG and the PPARγ agonist tesa was performed using standard DIC/HOBt activation (5 eq) for coupling and piperidine for Fmoc deprotection to obtain [K^4^(GFLG-tesa), F^7^, P^34^]-NPY (3).

All of the peptides were cleaved from the resin using TFA/scavenger (9:1 (v/v)) for 3 h, with TA/EDT (7:3, v/v) as a scavenger. The peptides were precipitated with ice cold diethyl ether, washed, and collected by centrifugation. RP-HPLC (Shimadzu) with a Kinetex column was used for peptide purification at a flow rate of 15 ml/min and a linear gradient system containing 0.1% (v/v) TFA in water (eluent A) and 0.08% (v/v) TFA in ACN (eluent B).

Pure products were characterized by analytical RP-HPLC, MALDI-ToF (Ultraflex III, MALDI-ToF/ToF, Bruker Daltonics), and ESI-HCT (high-capacity ion trap ESI-MS, Bruker Daltonics). For RP-HPLC, a LaChromeElite system (VWR) with Phenomenex Jupiter Proteo C12 90 Å and Agilent VariTide RPC columns was used with different linear gradients of eluent B (0.08% (v/v) TFA in ACN) in eluent A (0.1% (v/v) TFA in H_2_O), detection at 220 nm, and peak integration using EZ Chrome Elite software. The peptides were dissolved in DMSO with a final concentration of less than 0.1% DMSO for all of the in vitro experiments.

### 2.3 Cell culture

COS-7 (African green monkey kidney) cells stably expressing NPY_1/2/4/5_R and chimeric G protein (Δ6Gα_qi4-myr_) were cultured in DMEM High Glucose Medium with 10% (v/v) heat inactivated FCS, 100 units/ml penicillin, and 100 μg/ml streptomycin. HEK293 (human embryonic kidney) cells were grown in DMEM High Glucose Medium and Ham's F12 (1:1 (v/v)) supplemented with 15% (v/v) heat inactivated FCS. HEK293 cells stably expressing NPY_1/2/4/5_R C-terminally fused to eYFP were maintained in DMEM High Glucose Medium and Ham's F12 (1:1 (v/v)) supplemented with 15% (v/v) heat inactivated FCS and 100 μg/ml hygromycin B. 3T3-L1 (murine fibroblast) cells were grown in DMEM High Glucose Medium and 10% (v/v) not-heat inactivated FCS. The,3T3-L1 cells were split every second day to avoid confluency. SGBS (human fibroblast) cells were cultivated in DMEM High Glucose Medium and Ham's F12 (1:1 (v/v)) supplemented with 15% (v/v) not-heat inactivated FCS, 8 mg/l biotin and 4 mg/l pantothenate. The SGBS cells were split every third day to avoid confluency. All of the cell lines were maintained under a humidified atmosphere at 37 °C and 5% CO_2_.

### 2.4 Y-receptor activation studies

Signal transduction Ca^2+^-flux assays were performed as previously described [49]. Briefly, COS-7 cells stably expressing NPY_1/2/4/5_R and chimeric G protein (Δ6Gα_qi4*myr_) were seeded into black 96-well plates and grown for 24 h. The cells were incubated with 0.01% (v/v) Pluronic Acid F-127 and 2.4 μM Fluo2-AM in assay buffer (HBSS, 1.25 mM Probenecid, and 20 mM HEPES) at 37 °C for 60 min. A one-addition protocol of FlexStation 3 (Molecular Devices, San Jose, CA, USA) was used to perform fluorescent detection (excitation: 485 nm, emission: 525 nm). Indicated concentrations of peptides were added after a 20 s baseline recording, followed by 80 s detection of Ca^2+^ efflux. The signal response was quantified as x-fold over basal and normalized to the maximum response of the native ligand NPY (NPY_1/2/5_R) or hPP (NPY_4_R) set to 100%. The values were calculated using GraphPad Prism 5.0 via non-linear regression and represented the total mean ± SEM of the EC50 determined in n ≥ 2 independent experiments each performed in duplicate. The pEC50 ± SEM values corresponded to the negative decadic logarithm of the EC50 value.

### 2.5 Live cell microscopy

Y-receptor internalization was investigated as previously described [50]. Briefly, HEK293 cells stably expressing NPY_1/2/4/5_R C-terminally fused to eYFP were seeded into 8-well μ-slides and allowed to attach for 24 h. The cells were starved in Opti-MEM reduced serum medium (containing 100 μg/ml CHX and 6 μg/ml BFA) supplemented with Hoechst 33342 nuclear stain (0.5 mg/ml) at 37 °C for 30 min. The cells were then stimulated with 100 nM of peptide at 37 °C for 60 min and washed with acidic wash solution and HBSS. To visualize the peptide uptake, the cells were incubated with 100 nM TAMRA labeled [K4(GFLG-TAMRA), F^7^, P^34^]-NPY for 10 min, followed by an acidic wash (50 mM glycine, 180 mM NaCl, and pH 3.1) to remove the excess labeled peptide, washed in HBSS, and returned to the Opti-MEM. Microscopy images were taken using an Axio Observer microscope equipped with an ApoTome imaging system and a Heating Insert P Lab-Tek S1 unit (Zeiss, Oberkochen, Germany). Image editing was performed with AxioVision software version 4.6. The assays were performed in at least two independent experiments.

### 2.6 PPAR reporter gene assay

HEK293 cells stably expressing NPY_1_R C-terminally fused to eYFP were seeded into white 96-well plates, grown to 70–80% confluence, and transfected using Lipofectamine 2000 transfection reagent according to the manufacturer's protocol. For luciferase reporter gene assays, 250 ng reporter plasmid, positive control plasmid or negative control plasmid (Cignal PPAR Reporter (luc) Kit), 250 ng PPARγ expression vector, and 250 ng RXRα expression vector were applied in addition to 0.75 μl of Lipofectamine 2000 transfection reagent per well. For selectivity assays, HEK293 cells without stable NPY_1_R-eYFP transfection and HEK293 NPY_1_R-eYFP cells without PPARγ transfection were used. After 24 h of transfection, the cells were treated with tesa or peptides for an additional 24 h. For competition assays, the cells were treated with a 20-fold concentration of NPY simultaneous to peptide stimulation. Luciferase assays were performed using the Dual-Luciferase System Kit according to the manufacturer's instructions. The relative firefly luciferase activity was normalized by the corresponding Renilla luciferase activity, the luciferase activity of the cells transfected with the negative control plasmid, and the DMSO vehicle control. The assays were performed in at least three independent experiments.

### 2.7 Differentiation and AdipoRed assay of 3T3-L1 and SGBS cells

3T3-L1 or SGBS cells were seeded into 96-well plates and grown to complete confluency (set as day 0). On day 2 of confluence, the 3T3-L1 cells were differentiated by DMEM High Glucose Medium supplemented with 10% (v/v) not-heat inactivated FCS, 0.5 mM IBMX, 167 nM human insulin, and 1 μM dexamethasone (differentiation medium). On day 4, the medium was replaced by insulin medium (DMEM High Glucose Medium, 10% not-heat inactivated FCS, and 167 nM human insulin). On day 6, the medium was changed to standard culture medium and changed every other day until full differentiation was achieved (on day 8). The SGBS cells were differentiated as previously described [51]. Briefly, DMEM High Glucose Medium and Hams F12 (1:1 (v/v)) without FCS, supplemented with 0.01 mg human apo-transferrin, 20 nM human insulin, triiodo-l-thyronine, 100 nM hydrocortisone, 25 nM dexamethasone, 0.5 mM IBMX, and 2 μM rosiglitazone (quick differentiation medium, QD) was added on day 0. On day 4, the medium was replaced by 3FC medium (DMEM High Glucose Medium and Hams F12 (1:1 (v/v))) without FCS and supplemented with 0.01 mg human apo-transferrin, 20 nM human insulin, triiodo-l-thyronine, and 100 nM hydrocortisone. The medium was changed every second day until full differentiation was achieved (day 10).

On day 2 of the 3T3-L1 or SGBS differentiation process, tesa or peptides were added to the differentiation or QD medium at the indicated concentrations. For the SGBS, QD medium without rosiglitazone was used. After 24 h, differentiation was continued as previously described. To determine the intracellular triglyceride content as an indicator of adipocyte differentiation, the AdipoRed Adipogenesis Assay was performed according to the manufacturer's protocol. Fluorescence (excitation: 485 nm, emission: 572 nm) was measured with an Infinite 200 microplate reader (Tecan Group, Männedorf, Switzerland). For normalization, the relative differentiation was calculated as the DMSO vehicle control was set to 100%. Three independent experiments with four replicates were conducted.

### 2.8 qRT-PCR

The 3T3-L1 or SGBS cells were seeded into 6-well plates and differentiated as previously described. Tesa or peptides were added to the differentiation medium at a concentration of 100 μM on day 2, followed by 12 h or 24 h incubation. On day 4, the cells were washed two times with PBS, harvested by trypsinization, and stored at −70 °C until RNA isolation was performed. RNA extraction was performed using the RNeasy Mini Kit or TRIzol (Life Technologies, Grand Island, NY, USA), followed by reverse transcription with standard reagents (Life Technologies, Grand Island, NY, USA) or the QuantiNova Reverse Transcription Kit. qRT-PCR was performed with QuantiTect Primer Assays (Quiagen, Hilden, Germany) or TaqMan Gene Expression Assays (Thermo Fischer Scientific, Waltham, MA, USA: 36B4 #MM00725448, 18sRNA #Hs99999901, Fabp4 #MM00445878, adiponectin #MM00456425, and PPAR-gamma #MM00440940) using a Power SYBR Green or Brilliant SYBR Green QPCR Core Reagent Kit from Stratagene (La Jolla, CA, USA) according to the manufacturer's instructions on an Applied Biosystems 7500 Real-Time PCR or comparable system (Applied Biosystems, Foster City, CA, USA). N ≥ 3 independent experiments with two replicates were performed. For the cell culture experiments, the data were analyzed according to the 2^−ΔΔCt^ method using 36B4 or GAPDH as housekeeping genes and DMSO treatment as a control. Liver mRNA expression was calculated relative to 18sRNA, which was used as an internal control due to its resistance to glucose-dependent regulation [52]. The mRNA levels were quantified using the standard curve method of QuantStudio 6 Flex software (Applied Biosystems, Foster City, CA, USA), determining the crossing points of the individual samples using an algorithm that identifies the first turning point of the fluorescence curve. Amplification of the specific transcripts was confirmed by the melting curve profiles (cooling the sample to 68 °C and heating slowly to 95 °C while measuring the fluorescence) at the end of each PCR [53].

### 2.9 Animal studies

All of the experiments were performed according to the animal ethical laws of the state Saxony, Germany, and were approved by the local animal ethics review board (Landesdirektion Sachsen, Leipzig, Germany). Female *db/db* mice 12–15 weeks of age were purchased from Taconic (Denmark) and housed in groups of 5 in temperature- and humidity-controlled facilities in a 12 h:12 h light–dark cycle and had free access to tap water and food (regular chow, Sniff, Soest, Germany). Three out of seven groups served as controls (N = 15, see Table 1). The 5 db/db mice were untreated and 10 db/db mice were vehicle treated either orally or intraperitoneally (i.p.). One additional group (n = 5) of lean C57BL/6NTac mice was used as a metabolically healthy control group. Groups 1 to 5 were treated daily with 2.5 μmol/kg body weight tesa, peptides or vehicle (1% (v/v) DMSO in PBS) either by gavage or intraperitoneal injection for 8 days according to Table 1. The control mice were gavaged with an equal volume of vehicle.

**Table 1:** Groups of mice that were used for the in vivo Tesa-NPY studies.

Group	Mice	Treatment	Administration
1 (n = 5)	*db/db*	Tesa	Oral
2 (n = 5)	*db/db*	Tesa-NPY (3)	Intraperitoneal
3 (n = 5)	*db/db*	[F^7^, P^34^]-NPY (2)	Intraperitoneal
4 (n = 5)	*db/db*	Vehicle control	Oral
5 (n = 5)	*db/db*	Vehicle control	Intraperitoneal
6 (n = 5)	*db/db*	Untreated control	–
7 (n = 5)	C57BL/6N	Untreated healthy	–

### 2.10 Phenotypical characterization

All of the mice were monitored for 9 days during the treatment period. Body weight, food intake, and water uptake were recorded daily and body composition (lean body mass and whole body fat mass) was recorded at the beginning, middle, and end of the study in the conscious mice using an EchoMRI system (Echo Medical Systems, Houston, TX, USA). At the beginning and end of the treatment period, the HbA1c levels were determined from 5 μl of whole venous blood samples using an automated chemical analyzer at the Institute of Laboratory Medicine and Clinical Chemistry and beta-ketone using an automated glucose monitor (FreeStyle Precision H, Abbott GmbH, Ludwigshafen, Germany). Rectal body temperature was measured once on day 9 using Thermalert (TH-5, Physitemp, Clifton, NJ, USA). The mice were sacrificed on day 9 via an overdose of anesthetic (isoflurane, Baxter, Unterschleiβheim, Germany). Subcutaneous (sc) and epigonadal (epi) adipose tissue (AT) was immediately removed and frozen in liquid nitrogen. Serum was collected for measurements of triglycerides (TG), free fatty acidy (FFA), cholesterol, insulin, adiponectin, leptin, and monocyte chemotactic protein (Mcp-1).

### 2.11 Serum parameter analysis

Serum insulin (mouse insulin ELISA, Mercodia, Uppsala, Sweden), adiponectin (mouse adiponectin ELISA, AdipoGen, San Diego, CA, USA), leptin (mouse leptin ELISA, Crystal Chem, Downers Grove, IL, USA), and Mcp-1 (mouse/rat CCL2/JE/Mcp-1 Quantikine ELISA, R&D Systems, Minneapolis, MN, USA) levels were analyzed via ELISA according to the manufacturer's instructions. Serum concentrations of TG, FFA, and cholesterol were measured by an automatic chemical analyzer at the Institute of Laboratory Medicine and Clinical Chemistry.

### 2.12 Histology and adipocyte size measurements

Subcutaneous AT and epigonadal AT were fixed, paraffin embedded, sectioned (5 μm), and H&E stained as previously described [54]. Microscopy images were taken using an Axio Observer microscope (Zeiss, Oberkochen, Germany). The adipocyte size was analyzed from at least 50 cells per slice using AxioVision software version 4.6.

### 2.13 Microarray experiments and analyses

Transcriptome profiling was performed in epigonadal samples from 3 mice from each experimental group using a mouse Clariom S Assay from Affymetrix. The RNA integrity and concentration, RNA hybridization, scanning procedures, and post-processing were performed according to Affymetrix's protocol at our genetic technologies core unit. An expression matrix from the Affymetrix data was created using the robust multi-array average algorithm (RMA) in RMAExpress [55]. Briefly, the raw intensity values were background corrected, log2 transformed, and then quantile normalized. A linear model was then fit to the normalized data to obtain the expression measurements for each probe set on each array. Mapping of the manufacturer's Probe ID to data from public repositories (ENTREZ gene identifiers and ENSEMBL accession numbers) was performed using annotation packages available from Bioconductor version 3.7 (MacDonald JW (2017) clariomsmousetranscriptcluster.db: Affymetrix clariomsmouse annotation data (chip clariomsmousetranscriptcluster) using R package version 8.7.0). Gene expression data were analyzed using the R statistical environment (R Core Team (2017), R Foundation for Statistical Computing, Vienna, Austria, https://www.R-project.org). Log2-transformed values were analyzed using the Bayesian moderated t-statistic implemented in the limma package version 3.32.10 [56]. The linear model was fit to a design matrix including treatment as a factor variable with 2 levels and a “toptable” was produced with information on the fold changes in the gene expression according to the treatment, corresponding p-values, and multiple testing adjusted p-values according to the Benjamini-Hochberg procedure.

Gene ontology enrichment analysis for processes, functions, and components was performed using the Gene Ontology enRIchment anaLysis and visuaLizAtion tool (GOrilla) [57]. KEGG pathways and gene ontology (GO) gene sets were generated using kegg.gsets, go.gsets, and gageData. KEGG pathways were plotted using the Pathview package [58]. The figures were produced using the ggplot2 package [59], Pheatmap (Raivo Kolde (2016), Pheatmap: Pretty Heatmaps, R package version 1.0.9.), and REVIGO [60].

### 2.14 Statistical analysis

Statistical analysis was performed using GraphPad Prism 5.03 (GraphPad Software, Inc., San Diego, CA, USA). Statistical significance was determined via one-way analysis of variance (ANOVA) followed by Dunnett's multiple comparison test for the cell culture studies and the non-parametric Mann–Whitney U-test for the animal studies. Statistical analyses for microarray data were previously described.

## 3 Results

### 3.1 Peptide synthesis of Tesa-NPY

**Figure 2:**
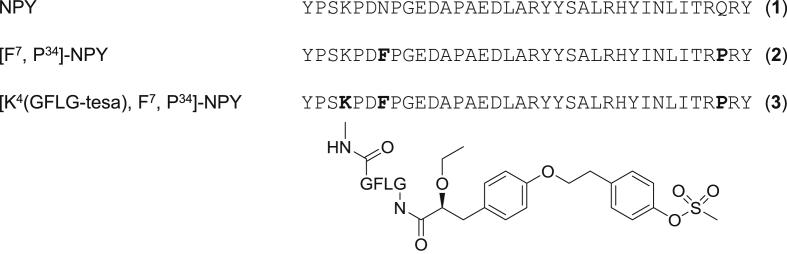
**Sequences and structures of peptides.** Peptide sequences of NPY (1), [F^7^, P^34^]-NPY (2), and [K^4^(GFLG-tesa), F^7^, P^34^]-NPY (3). Chemical structures of the PPARα/γ agonist tesa and the cleavable GFLG linker used to attach tesa to the peptide.

Unmodified NPY (1) and NPY_1_R-preferring ligands [F^7^, P^34^]-NPY (2) were synthesized by automated solid phase peptide synthesis (SPPS) using the orthogonal Fmoc/tBu strategy [61]. For [K^4^(GFLG-tesa), F^7^, P^34^]-NPY (tesa-NPY) (3), a combination of automated SPPS and manual couplings was used. Tesa was then linked to [F^7^, P^34^]-NPY (2) by an enzymatically cleavable GFLG linker (Figure 2).

**Figure 3:**
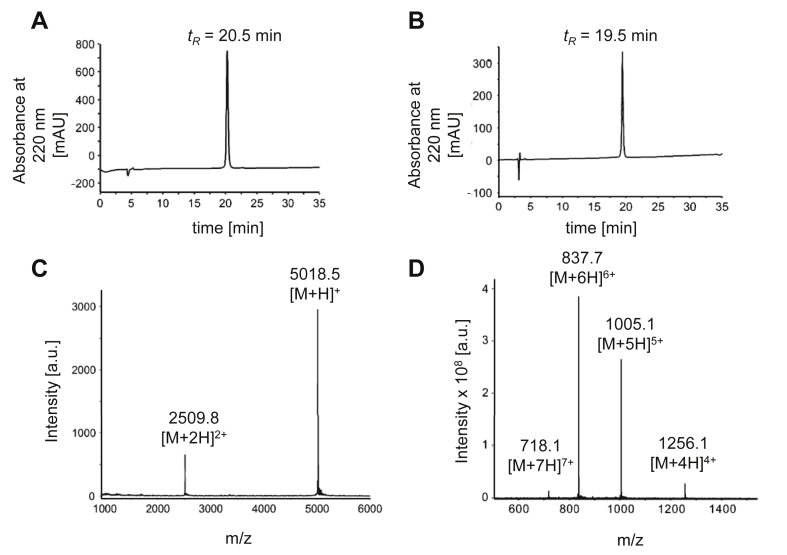
**Analytical data of [K**^**4**^**(GFLG-tesa), F**^**7**^**, P**^**34**^**]-NPY (3).** (A) Analytical RP-HPLC using a Phenomenex Jupiter Proteo C12 90 Å column and (B) an Agilent VariTide RPC column with a linear gradient of 30–60% (v/v) ACN/H_2_O in 30 min. (C) MALDI-ToF mass spectrum showing the single- and double-loaded molecule ion signals (M_calc._ = 5017.5 Da). (D) ESI mass spectrum displaying the four-, five-, six-, and seven-fold charged molecule ions. tR = retention time, AU = absorption units, a.u. = arbitrary units.

Previous studies demonstrated that modifications at position 4 of NPY do not change the peptide's activation and internalization behavior [[62], [63], [64]]. Modification of the *Nε* group of Lys^4^ was achieved by the selective hydrazine-induced removal of the orthogonal Dde protecting group and the subsequent attachment of the GFLG linker and tesa via standard DIC/HOBt coupling. After cleavage from the Rink amide resin, all of the peptides were purified by RP-HPLC to a purity of >95%. The identity and purity of the peptides were confirmed by MALDI-ToF, ESI-HCT mass spectrometry, and analytical RP-HPLC (Table 2). The analytical data for tesa-NPY (3) are shown in Figure 3.

**Table 2:** Analytical characterization of the synthesized peptides.

No.	Compound	MALDI-ToF MS		ESI	RP-HPLC retention time (min)		Purity (%)
		M_calc._ (Da)	M_obs._ (M + H)^+^	M_obs._ (z) (Da)	Column A	Column B	
(1)	NPY	4251.1	4252.0	1064.3 (4+) 851.6 (5+) 709.8 (6+) 608.5 (7+)	22.0 (20-60-40)	18.7 (20-60-40)	>95
(2)	[F^7^, P^34^]-NPY	4253.1	4254.0	1064.9 (4+) 852.0 (5+) 710.9 (6+) 609.0 (7+)	23.3 (20-60-40)	19.8 (20-60-40)	>95
(3)	[K^4^(GFLG-tesa), F^7^, P^34^]-NPY	5017.5	5018.5	1256.1 (4+) 1005.0 (5+) 837.7 (6+) 718.1 (7+)	20.5 (30-60-30)	19.5 (30-60-30)	>95

### 3.2 Tesa-NPY is a potent NPY_1_R agonist and induces NPY_1_R internalization

**Figure 4:**
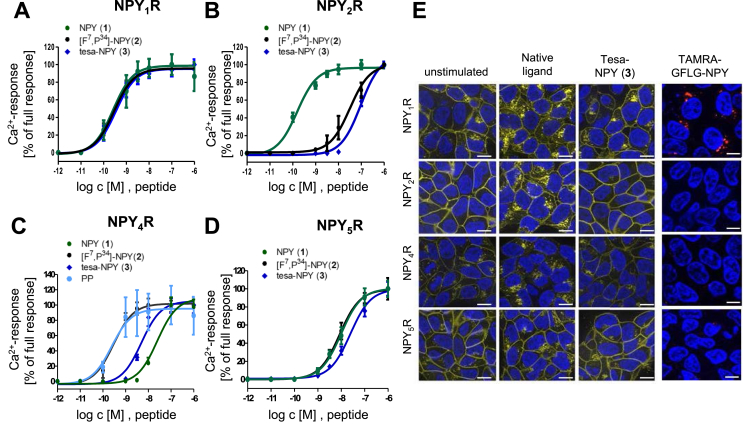
**Receptor activation and internalization of peptides.** (A–D) Ca^2+^-flux assays in stably transfected COS-7 cells to measure the activity of NPY (1) or hPP, [F^7^, P^34^]-NPY (2), and tesa-NPY (3) on the respective human Y-receptor subtype NPY1R (A), NPY_2_R (B), NPY_4_R (C), NPY_5_R (D). The depicted values represent the mean ± SEM from n ≥ 2 independent experiments. Data were normalized to maximum NPY (NPY_1/2/5_R) or hPP (NPY_4_R) response. (E) Receptor internalization of (1) and (3) studied in HEK293 cells stably expressing the respective human Y-receptor subtype fused to eYFP (yellow). Cells were stimulated with 100 nM of peptides for 1 h. Uptake of the fluorescent-labelled linker peptide [K^4^(GFLG-TAMRA), F^7^, P^34^]-NPY (red) was monitored after incubation for 10 min Hoechst33342 was used for nuclear staining (blue). Representative pictures from two independent experiments. Scale = 10 μm.

The receptor activation, selectivity, and internalization were investigated to ensure that the attachment of the cleavable linker and tesa did not alter the behavior of NPY_1_R-preferring carrier peptide [F^7^, P^34^]-NPY. The activation of the human Y-receptors was tested using Ca^2+^-flux assays in COS-7 cells stably expressing one specific Y-receptor subtype (NPY_1/2/4/5_R) and chimeric G protein (Δ6Gα_qi4-myr_), opening the Ca^2+^ channels upon receptor activation (Figure 4A-D, Table 3) [49,65].

**Table 3:** Receptor activation of peptides determined via Ca^2+^-flux assay.

No.	Peptide	EC_50_ (nM) (pEC_50_ ± SEM)			
		NPY_1_R	NPY_2_R	NPY_4_R	NPY_5_R
(1)	NPY/hPP	0.4 (9.4 ± 0.2)	0.2 (9.8 ± 0.1)	0.2 (9.6 ± 0.4)	9.9 (8.0 ± 0.1)
(2)	[F^7^, P^34^]-NPY	0.3 (9.5 ± 0.1)	32 (7.5 ± 0.1)	0.3 (9.5 ± 0.1)	8.5 (8.0 ± 0.1)
(3)	tesa-NPY	0.4 (9.4 ± 0.1)	96 (7.0 ± 0.1)	5.0 (8.3 ± 0.1)	25 (7.6 ± 0.1)

Tesa-NPY (3) is a potent NPY_1_R agonist as indicated by EC_50_ values comparable to that of carrier peptide [F^7^, P^34^]-NPY (2) and native ligand NPY (1). At NPY_2_R, a 400-fold loss in the tesa-NPY (3) activity was observed compared to NPY (1), which was even higher than the 160-fold loss of NPY_1_R-preferring [F^7^, P^34^]-NPY (2). These data demonstrate the strong NPY_1_R selectivity of the peptide-drug conjugate toward NPY_2_R. The activity of [F^7^, P^34^]-NPY (2) at NPY_4_R and NPY_5_R was comparable to the activity of the native ligands hPP (NPY_4_R) and NPY (NPY_5_R) (1). This limited selectivity of [F^7^, P^34^]-NPY toward NPY_4_R and NPY_5_R was previously detected during its development [54,58]. However, the modification of [F^7^, P^34^]-NPY with tesa led to a strong loss of activity (25-fold) at the NPY_4_R subtype and a slight loss of activity at NPY_5_R. These results demonstrate that the tesa-modified [F^7^, P^34^]-NPY (3) was more NPY_1_R selective than the unmodified [F^7^, P^34^]-NPY (2), especially with respect to the effects at NPY_4_R.

The selectivity of tesa-NPY (3) was further investigated by live cell imaging of ligand-induced Y-receptor internalization using a HEK293 cell line that stably expresses NPY_1/2/4/5_R-eYFP fusion protein (Figure4E). The receptors (yellow) were mainly present in the membrane prior to stimulation and only minor fluorescence was detected in the intracellular compartments, which was caused by overexpression in the stable cell lines and due to the accumulation of receptor proteins in the endoplasmic reticulum and Golgi apparatus [62,66,67]. Because we observed continuous replenishment of membrane-localized NPY_1_R and NPY_4_R receptors during internalization, we added translation inhibitor CHX and ER-Golgi transport inhibitor BFA to the medium to facilitate the detection of reduced receptor localization to the membrane upon internalization. The native ligand NPY (1) and peptide conjugate tesa-NPY (3) induced NPY_1_R internalization as indicated by an increase in the yellow fluorescence in the intracellular vesicles and a decrease in the membrane fluorescence. For NPY_2_R and NPY_4_R, only the native ligands NPY (NPY_2_R)/hPP (NPY_4_R) induced an internalization, whereas stimulation with tesa-NPY (3) led to only minor endocytosis. For NPY_5_R, it was previously described that internalization occurs much slower and in a lower amount than for the other Y-receptor subtypes, which was also detected in the present study [68]. To confirm these findings, we incubated the cells with fluorescently labeled [K^4^(GFLG-TAMRA), F^7^, P^34^]-NPY and monitored the uptake of red fluorescent linker peptide used to shuttle tesa preferably to NPY_1_R-expressing cells.

These results demonstrated that NPY_1_R activity and the preference of tesa-NPY (2) over NPY_2_R and NPY_4_R was even higher than that of unmodified [F^7^, P^34^]-NPY (3). Selectivity toward NPY_5_R can be ensured as this receptor undergoes only minor endocytosis.

### 3.3 Tesa-NPY induces PPARγ transcriptional activity in cells expressing NPY_1_R and PPARγ

**Figure 5:**
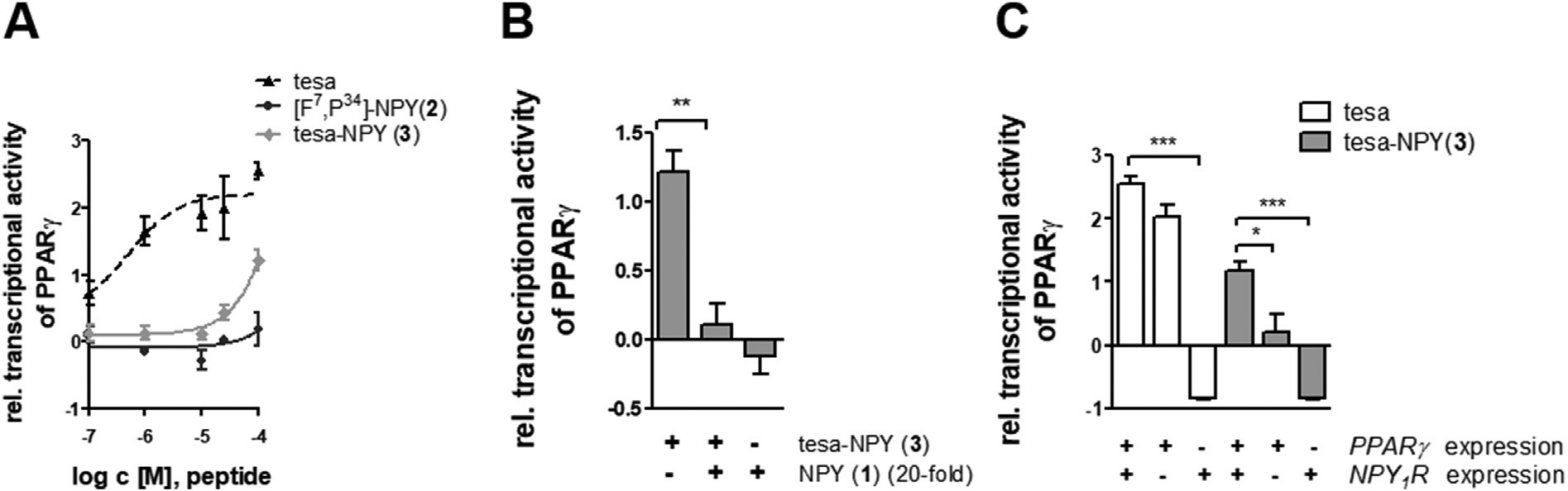
**Transcriptional activity of the PPAR reporter gene.** (A) Relative transcriptional activity following tesa, [F^7^, P^34^]-NPY (2), or tesa-NPY (3) treatment in HEK293 cells stably expressing NPY_1_R, transiently transfected with PPAR reporter gene, PPARγ, and RXRα plasmids. (B) Relative transcriptional activity following treatment with 100 μM of tesa-NPY (3) in the presence or absence of 20-fold NPY (1) using HEK293 cells stably expressing the NPY_1_R, transiently transfected with the PPAR reporter gene, PPARγ and RXRα plasmids. Treatment with NPY (1) alone served as a negative control. (C) Relative transcriptional activity following treatment with 100 μM tesa or tesa-NPY (3) in HEK293 cells expressing either NPY_1_R or PPARγ or both (in the presence of PPAR reporter gene- and RXRα expression plasmids). The depicted values represent mean ± SEM from n ≥ 3 independent experiments. All measurements were normalized to the transcriptional activity of DMSO-treated cells expressing all factors (set to 0). Statistical significance was determined by one-way analysis of variance (ANOVA) followed by Dunnett's multiple comparison test, *≤0.05, **p ≤ 0.01, ***p ≤ 0.001.

To examine whether the tesa-NPY conjugate (3) was able to activate the PPARγ nuclear receptor, HEK293 cells stably expressing NPY_1_R were transfected with a plasmid-expressing luciferase under the control of the PPAR-responsive element PPRE. Furthermore, plasmids coding for PPARγ and RXRα were transfected. Because PPARγ binds as a heterodimer with RXRα to PPRE-responsive elements, RXRα is needed for PPARγ activity [40]. After transfection, the cells were treated with free tesa, carrier peptide [F^7^, P^34^]-NPY (2), or the tesa-NPY conjugate (3) and the luciferase activity was measured (Figure 5).

Concentration-dependent luciferase activity was detected in the tesa and tesa-NPY (3) treated cells (Figure5A). [F^7^, P^34^]-NPY (2), in contrast, did not contain a PPARγ agonist and was thus unable to induce transcriptional activity. However, the free tesa demonstrated a saturation curve, whereas saturation was not achieved for the tesa-NPY (3). Most likely, higher concentrations of the drug are needed for comparable effects. However, with respect to higher concentrations, the assay is technically limited due to the peptide's solubility. The peptide's lower potency was likely due to the selective transport and release of the peptide conjugate. Whereas free tesa is taken up by the cells through passive diffusion, peptide conjugate needs to be taken up by endocytosis and the drug has to be released. This process is further limited by the receptor expression level on the cell surface. Thus, higher concentrations of peptide conjugate are needed to reach the same concentration inside the cell, as was previously shown for cytotoxic NPY conjugates with comparable size and polarity [63,66].

To confirm that the PPARγ activity of the tesa-NPY (3) was induced by NPY_1_R-mediated internalization, the effect of the conjugate was investigated in the presence of an excess of unmodified NPY_1_R agonist (NPY) (Figure5B). Incubation with NPY alone did not lead to any transcriptional activity, whereas tesa-NPY induced it. Indeed, unlabeled NPY competed with tesa-NPY (3) for binding to NPY_1_R and therefore its transcriptional activity, confirming the receptor-mediated mechanism of PPARγ activation by the peptide-drug conjugate. Further verification of the peptide conjugate specificity was performed using cells that do not express either NPY_1_R or PPARγ (Figure5C). The activity of the free tesa, taken up by passive diffusion, was not influenced by NPY_1_R expression, whereas the absence of PPARγ led to a complete loss of transcriptional activity (left). In contrast, for the tesa-NPY conjugate (3), the presence of both the receptor and PPARγ was mandatory to exert its effects, as expected for receptor-mediated uptake of the compound. In the absence of NPY_1_R, the levels dropped to basal levels comparable to unstimulated PPARγ expressing cells. In the absence of PPARγ, even the basal levels of transcriptional activity were lost, leading to negative values in the assay. Apparently HEK cells do not express detectable levels of endogenous PPAR proteins that can be activated in the assay.

These results demonstrated that tesa was selectively internalized through [F^7^, P^34^]-NPY and activated PPARγ in the cells stably expressing NPY_1_R. Next, the activity of tesa-NPY in cells with native NPY_1_R expression is addressed.

### 3.4 Tesa-NPY enhances adipogenesis and the expression of adipocyte-marker genes in 3T3-L1 and SGBS cells

**Figure 6:**
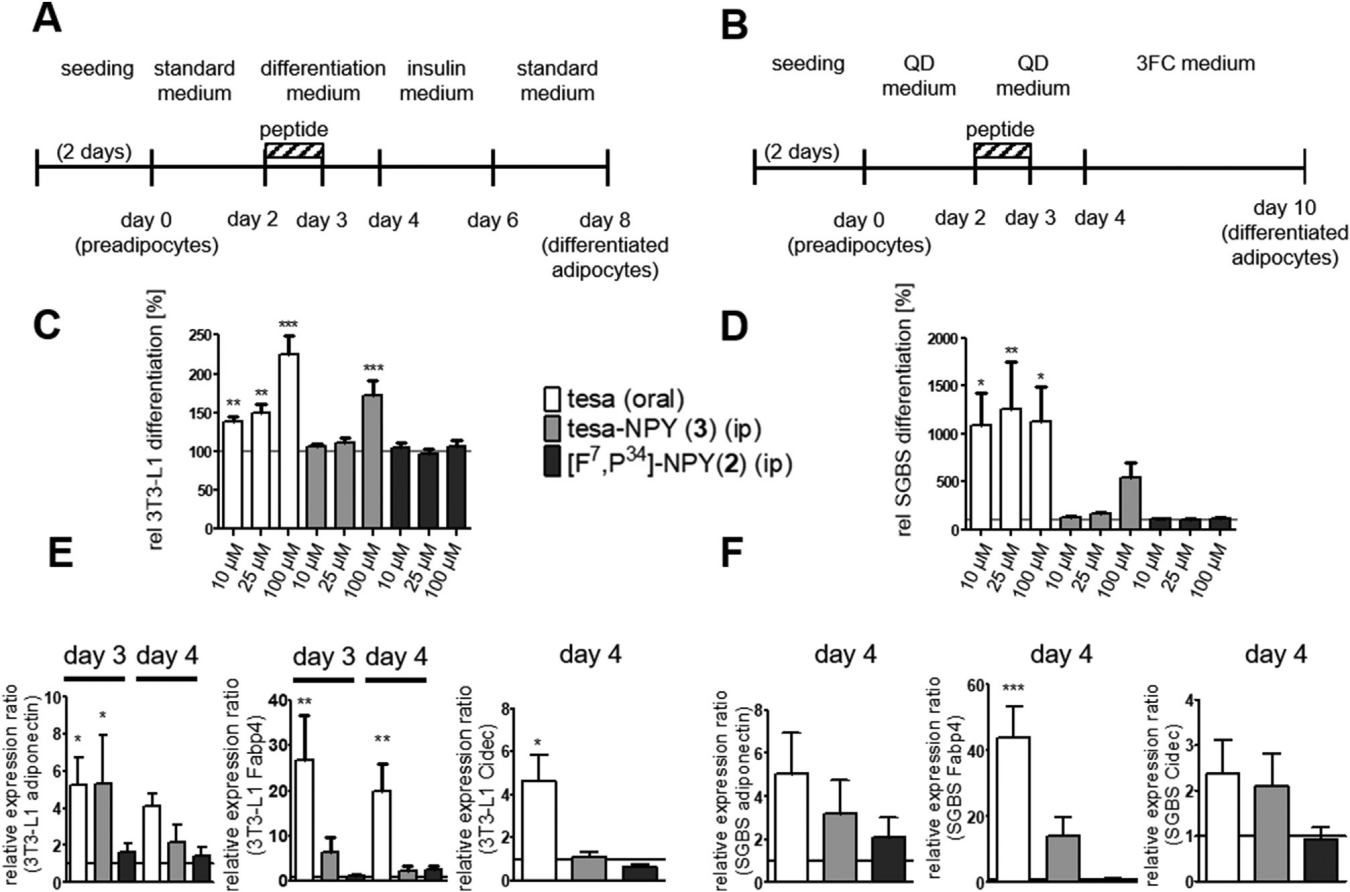
**Influence of tesa-NPY (3) on adipogenesis.** (A, B) Cells were differentiated and stimulated according to the protocol depicted for 3T3-L1 (A) and for SGBS (B). (C, D) Lipid accumulation was measured by TG staining in differentiated 3T3-L1 (C) or SGBS (D) adipocytes following treatment with indicated concentrations of tesa, [F^7^, P^34^]-NPY (2), or tesa-NPY (3). Bars represent mean ± SEM from n = 3 independent experiments performed in quadruplicates. Measurements were normalized using DMSO (vehicle)-treated cells (set to 100%). Statistical significances refer to vehicle-treated cells. (E,F) Expression of adipocyte-enriched genes in 3T3-L1 (E) or SGBS (F) adipocytes analyzed by qPCR. Values represent mean ± SEM from n ≥ 3 independent experiments performed in duplicates. Measurements were normalized according to the 2^−ΔΔCt^ method to 36B4 or GAPDH as housekeeping genes and DMSO-treated cells as control (set to 1). Statistical significances refer to DMSO-treated cells and were determined by one-way analysis of variance (ANOVA) followed by Dunnett's multiple comparison test, *≤0.05, **p ≤ 0.01, ***p ≤ 0.001.

The activation of PPARγ is sufficient and necessary for adipogenesis [69]. Thus, the tesa-NPY conjugate (3) was analyzed for its ability to stimulate murine 3T3-L1 and human SGBS preadipocyte differentiation into mature adipocytes. This was assessed via quantification of the intracellular triglyceride (TG) content, as adipocytes accumulate TG in response to cell differentiation (Figure 6A-D) [70]. The 3T3-L1 and SGBS cells were differentiated as illustrated in Figure 6A/B. Treatment with 10/25/100 μM of free tesa and 100 μM of tesa-NPY (3) enhanced adipogenesis in both cell lines (Figure 6C/D). In contrast, [F^7^, P^34^]-NPY (2) had no influence on adipocyte differentiation. As previously discussed for the reporter gene assay in HEK cells (Figure 5), higher concentrations (unfeasible due to solubility issues) or longer constant treatment with tesa-NPY (3) is needed to obtain comparable effects as observed for free tesa.

Moreover, PPARγ activation leads to the expression of several adipogenic markers, such as adiponectin, fatty acid binding protein 4 (Fabp4), and cell death-inducing DEFA-like effector c (Cidec). The expression of these genes in response to tesa-NPY (3) was assessed via qPCR (Figure 6E/F) [[71], [72], [73], [74]]. The 3T3-L1 and SGBS cells were differentiated as previously described, harvested on day 3 or 4, and used for qPCR. The expression of adiponectin in the 3T3-L1 cells was upregulated by the free tesa and peptide conjugate (3), but not for [F^7^, P^34^]-NPY (2). The expression of Fabp4 and Cidec in the 3T3-L1 cells was enhanced by free tesa, but no significant effect of tesa-NPY (3) and [F^7^, P^34^]-NPY (2) was visible. In the SGBS cells, tesa and tesa-NPY (3) enhanced the expression of adiponectin, Fabp4, and Cidec. However, only Fabp4 induction by tesa reached significant differences. [F^7^, P^34^]-NPY (2) showed no effect.

These data point to successful internalization, release, and activity of tesa-NPY (3) in cells natively expressing NPY_1_R. Finally, we tested whether the peptide conjugate was active and able to induce anti-diabetic effects in vivo.

### 3.5 Tesa-NPY influences body weight and adipose tissue morphology in *db/db* mice

**Figure 7:**
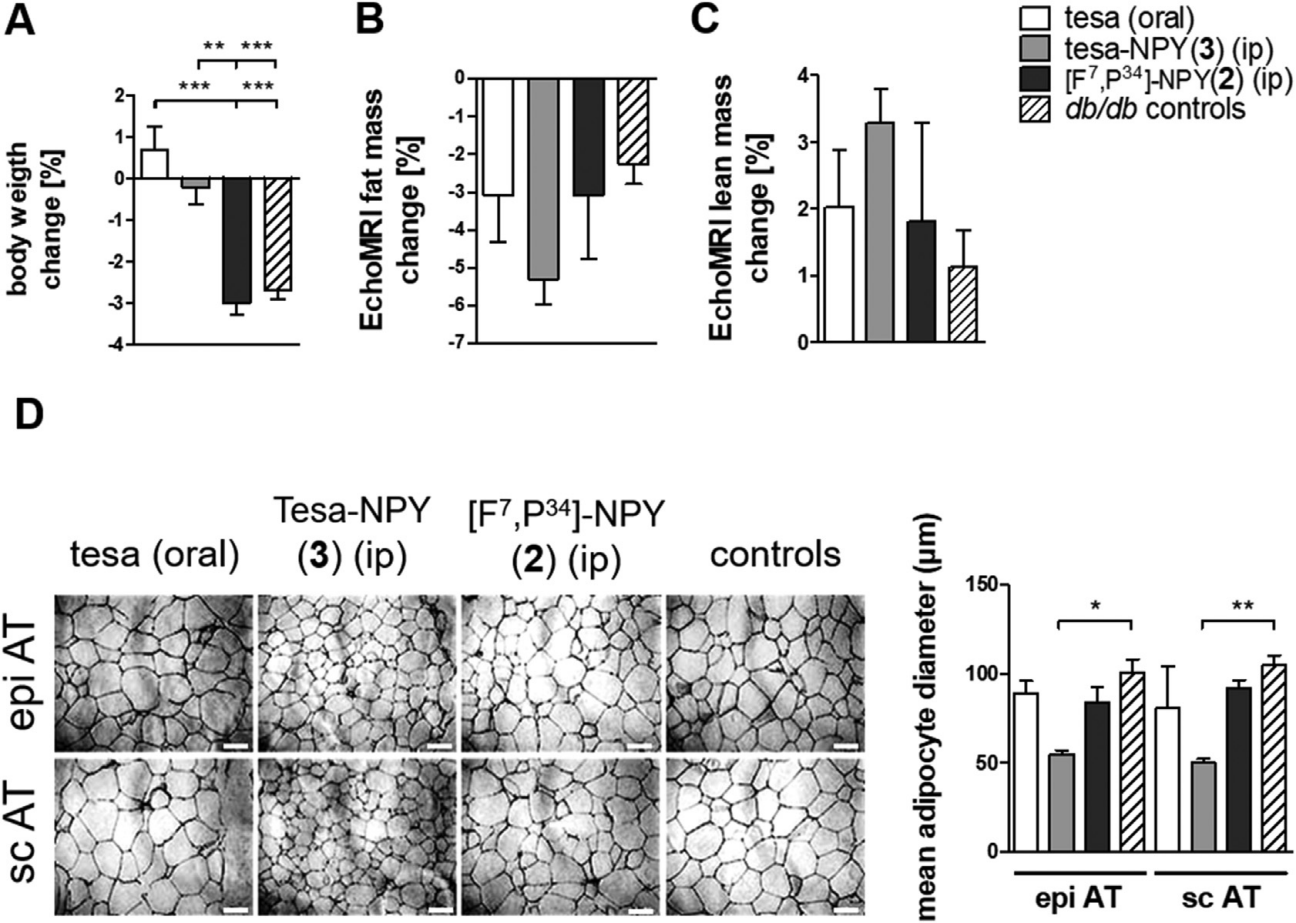
**Effects of tesa-NPY (3) on body weight, body composition and adipose tissue morphology in *db/db* mice.** (A) Percentage of body weight change over 9 days of mice treated with 2.5 μM/kg/day tesa, [F^7^, P^34^]-NPY (2), or tesa-NPY (3) (n = 5). (B, C) The percentage of fat (B) mass and lean (C) mass change determined by EchoMRI over 9 days of mice treated with tesa (2) or tesa-NPY(3) (n = 5). Bars represent mean ± SEM; * ≤0.05, **p ≤ 0.01, ***p ≤ 0.001 determined by one-way analysis of variance (ANOVA) followed by Dunnett's multiple comparison test. D) Adipose tissue morphology determined by H&E staining of epigonadal (epi) and subcutaneous (sc) adipose tissue (AT) of mice treated with tesa, (2), (3) or vehicle. Controls represent untreated mice and mice treated with vehicle (oral or intraperitoneal) (n = 15). Scale bar = 100 μM. Mean epi and sc adipocyte diameters were analyzed using the AxioVision software release 4.8.

The *db/db* mice were treated with 2.5 μM/kg/day tesa, tesa-NPY (3), or [F^7^, P^34^]-NPY (2) over 8 days. The controls represent the untreated *db/db* mice and the *db/db* mice treated with vehicle (1% (v/v) DMSO in PBS). Changes in body weight during the treatment were measured. The mice treated with tesa and tesa-NPY (3) did not change significantly, whereas their littermates treated with [F^7^, P^34^]-NPY (2) or vehicle/untreated lost approximately 3% of their body weight (Figure 7A). However, no significant differences between the body composition of the mice (lean mass and fat mass determined by EchoMRI) were detected (Figure 7B/C).

To investigate whether treatment with tesa, tesa-NPY (3), or [F^7^, P^34^]-NPY (2) affected the adipocyte morphology, the adipose tissue histology was analyzed and the adipocyte size distribution was measured. H&E staining of the subcutaneous and epigonadal adipose tissue depots showed a trend toward smaller epigonadal and subcutaneous cell size in the mice treated with tesa-NPY (3), whereas the mean adipocyte diameter for all of the other mice was comparable (Figure 7D).

### 3.6 Tesa-NPY influences metabolic parameters in *db/db* mice

**Figure 8:**
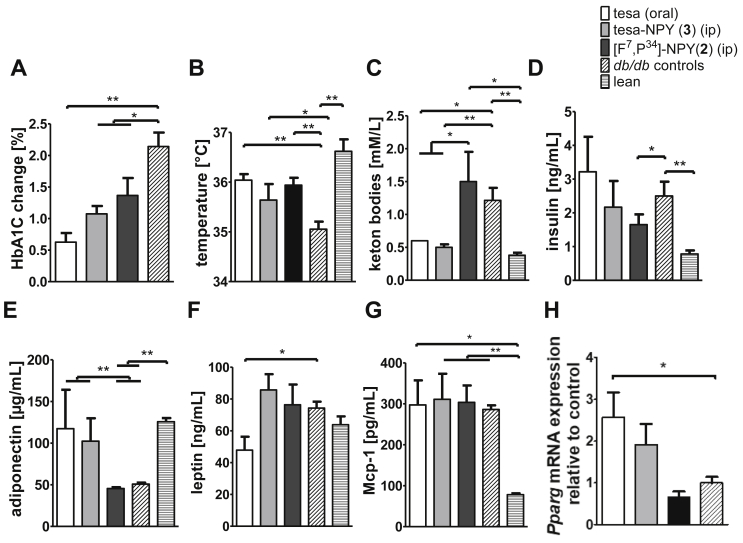
**Influence of tesa-NPY (3) on metabolic parameters in db/db mice.** (A–H) 2.5 μM/kg/day tesa, [F^7^, P^34^]-NPY (2), or tesa-NPY (3) was administered for 8 days in *db/db* mice (n = 5) and different metabolic parameters including serum HbA1C change(A), temperature (B), ketone bodies (C), serum insulin (D), serum adiponectin (E), serum leptin (F), and serum Mcp-1 (G) were analyzed. In addition, PPARγ mRNA expression in liver was quantified relative to control animals (H). Controls represent untreated mice and mice treated with vehicle (oral or intraperitoneal) (n = 15). Values represented as mean ± SEM; *p ≤ 0.05, **p ≤ 0.01, ***p ≤ 0.001, according to the non-parametric Mann–Whitney U test.

In addition to the body weight and body composition, several metabolic parameters were measured after treatment with tesa, tesa-NPY (3), [F^7^, P^34^]-NPY (2), or vehicle/untreated. The vehicle/untreated *db/db* mice had elevated plasma levels of HbA1C (data not shown), ketone bodies, insulin, and Mcp-1 and reduced plasma levels of adiponectin compared to the lean mice (Figure 8), which is characteristic of these mice. The treatment significantly delayed rapid diabetes progression, which is prototypical for *db/db* mice. Whereas in the vehicle/untreated mice, the HbA1C values increased by approximately 2%, a graduated reduced increase was seen for [F^7^, P^34^]-NPY (2), peptide conjugate (3), and tesa (Figure 8A). Body temperature, which decreased to 35 °C in the vehicle/untreated *db/db* controls, was normalized to 36 °C in all of the treated mice including the mice treated with [F^7^, P^34^]-NPY (2) (Figure 8B). Treatment with tesa and tesa-NPY (3) led to normalization of the plasma concentration of ketone bodies and adiponectin, whereas [F^7^, P^34^]-NPY (2) and vehicle/untreated showed no effect (Figure 8C/E). Treatment had no major influence on the insulin and Mcp-1 levels (Figure 8D/G). The serum leptin concentration was reduced in the mice treated with tesa, whereas no reduction was detectable in all of the other treated mice (Figure 8F). Only tesaglitazar significantly enhanced the expression of PPARγ in the liver (Figure 8H).

**Figure 9:**
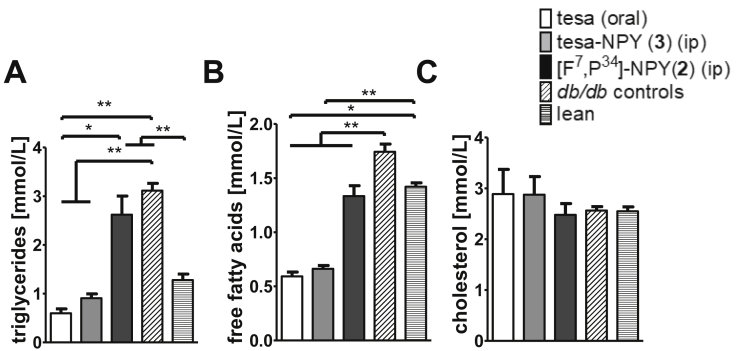
**Influence of tesa-NPY (3) on plasma lipids in *db/db* mice.** (A–C) 2.5 μM/kg/day tesa, [F^7^, P^34^]-NPY (2), or tesa-NPY (3) was administered for 8 days in *db/db* mice (n = 5) and triglycerides (A), free fatty acids (B), and cholesterol (C) were analyzed. Controls represent untreated mice and mice treated with vehicle (oral or intraperitoneal) (n = 15). Values represent mean ± SEM; *p ≤ 0.05, **p ≤ 0.01, ***p ≤ 0.001, according to the non-parametric Mann–Whitney U test.

The influence of tesa, tesa-NPY (3), and [F^7^, P^34^]-NPY (2) on the plasma lipids was also analyzed (Figure 9). The vehicle/untreated *db/db* mice showed elevated levels of triglycerides and free fatty acids (FFA) compared to the lean C57BL/6N mice. Treatment with tesa and tesa-NPY (3) led to a normalization of the triglycerides, FFA, whereas [F^7^, P^34^]-NPY (2) and vehicle/untreated had no influence on the lipid metabolism (Figure 9A/B). The cholesterol levels were unchanged by any treatment as these levels were also comparable in the untreated *db/db* mice compared to the lean mice (Figure 9C).

### 3.7 Microarray data analyses demonstrates differential expression of genes highly relevant to glucose metabolism and adipogenesis

**Figure 10:**
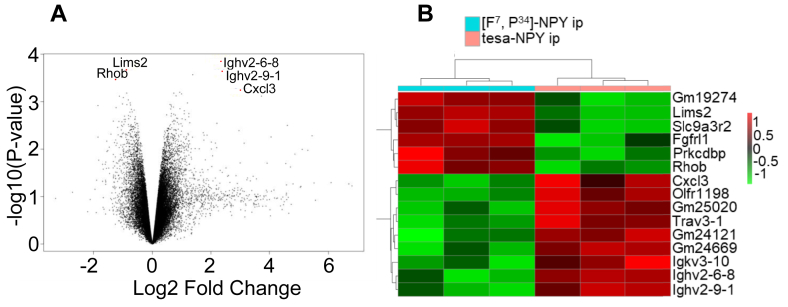
**Microarray data analyses revealed differentially expressed genes between animals treated with tesa-NPY and [F7, P34]-NPY.** (A) Volcano of differentially expressed genes in Tesa_NPY- vs FP_NPY-treated animals. RhoB (p = 0.00033), Lims2 (p = 0.000209), Fgfrl1 (0.000820), and Cxcl13 (p = 0.00057) are highly regulated genes. Transcriptome profiling was performed in epigonodal samples of 3 mice from each experimental group using mouse Clariom™ S Assay from Affymetrix. Raw intensities values were background corrected, log2 transformed and then quantile normalised. Log_2-_ transformed values were analyzed using the Bayesian moderated t-statistic. The linear model was fit to a design matrix including treatment as a factor variable with 2 levels and a “toptable” was produced with information on fold change in gene expression according to treatment, corresponding p-values and multiple testing adjusted p-values according to Benjamini Hochberg. (B) Heatmap with differentially regulated genes in Tesa_NPY- vs FP_NPY-treated animals with p-value < 0.001.

To identify the regulated novel genes and pathways, we measured the mRNA expression in the adipose tissue of the treated and control mice using a microarray approach. In line with morphological changes observed in adipose tissue and metabolism under tesa-NPY, microarray data analyses revealed differential increases and decreases of genes highly relevant to glucose metabolism and adipogenesis (Table S1, S2, Figure 10). The gene expression comparison of the animals treated with tesa-NPY compared to [F^7^, P^34^]-NPY identified ras homolog family member B (*RhoB*) (logFC = −1.23, p-value = 0.00033), lens intrinsic membrane protein 2 (*Lims2*) (logFC = - 0.87, p = 0.000209), fibroblast growth factor receptor-like 1 (*Fgfrl1*) (log2FC = - 0.9, p-value = 0.000820), C-X-C motif chemokine ligand 13 (*Cxcl13*) (logFC = 3.008, p-value = 0.00057), and the genes involved in antigen recognition s.a. Ighv2-6-8 and Ighv2-9-1 (Table S1 and S2) as highly regulated genes (Figure 10). The most significant enriched pathway of differentially expressed genes was related to lipid metabolism (fatty acid degradation and increased arachidonic and linoleic acid metabolism), the PPAR signaling pathway, drug metabolism in tesa-NPY treatment compared with FP-NPY, and insulin signaling (Table S3). Depleted pathways under tesa-NPY included cell cycle, cell turnover, apoptosis, and oxidative phosphorylation. Interestingly, the KEGG pathway for insulin signaling was downregulated although the gene ontology analyses of the biological processes and cell components showed enrichment of the gene sets involved in the positive regulation of glucose import and the negative regulation of the sequestering of triglycerides (Table S3).

## 4 Discussion

Because of its beneficial effects on glucose and lipid abnormalities in patients with type 2 diabetes, PPARα/γ agonist tesaglitazar is a promising candidate for clinical applications. However, severe side effects related to the essential role of PPAR in the regulation of numerous processes in a variety of cell types and tissues limit its therapeutic value. In this report, we describe the development of a system for the cell type-specific delivery of dual PPARα/γ agonist tesa into adipocytes. This strategy allows the use of PPARα/γ agonists to selectively drive adipocyte differentiation and avoid their adverse effects in other tissues that has hindered the clinical development of these compounds. Peptide-drug conjugates, prodrugs synthesized by covalent coupling of a peptide to a drug via a specific cleavable linker, enter the cells by specific receptor-mediated binding and internalization in the receptor-expressing cells. Subsequent endosomal cleavage of the linker releases the drug to freely diffuse in the cells and exert its effects. Peptide-drug conjugates were investigated as a promising approach for the selective delivery of cytotoxic agents to tumors in cancer therapy and the first compounds are currently being evaluated in clinical trials [75]. Initial attempts to use this principle for the treatment of T2D have been achieved by targeting estrogen to GLP-1R-expressing tissues such as the pancreas [76].

### 4.1 Design of drug-peptide conjugates

We utilized NPY_1_R as the cell surface receptor to selectively target adipocytes, as this GPCR was shown to be highly expressed in adipocytes and overexpressed in the adipose tissue of obese patients [[27], [28], [29], [30]]. NPY_1_R belongs to a multi-receptor multi-ligand family consisting of four receptor subtypes in humans (NPY_1_R, NPY_2_R, NPY_4_R, and NPY_5_R) that can be activated by NPY. Therefore, NPY_1_R subtype selectivity of the carrier peptide is of high importance and has to be ensured [77,78]. This can be achieved by using [F^7^, P^34^]-NPY, a NPY_1_R-preferring peptide ligand, which was already successfully used to target NPY_1_R-positive breast cancer cells [[61], [62], [63],^66^,^79^,^80^]. We synthesized tesa-NPY (3), a conjugate consisting of PPARα/γ agonist tesa attached to [F^7^, P^34^]-NPY through a cleavable GFLG linker introduced at position K^4^. Tesa-NPY activates high-activity NPY_1_R and robustly induces an internalization of the receptor. Furthermore, the investigation of the peptide conjugate among other human Y receptors demonstrated that tesa-NPY has very low activity at NPY_2_R and NPY_4_R. Interestingly, the modifications in the peptide conjugate led to a further reduction in activity at NPY_2_R and NPY_4_R compared to unmodified [F^7^, P^34^]-NPY. This might be crucial especially with respect to NPY_4_R, as this receptor is highly expressed in the gastrointestinal tract, pancreas, and prostate [81,82]. Selectivity toward cells expressing NPY_5_R can also be ensured, as these receptors showed only minor internalization and were mainly expressed in the CNS; thus, the effects at this receptor will be limited by the blood–brain barrier [[34], [35], [36],^80^,^83^].

### 4.2 Successful specific peptide-mediated shuttling of tesa to NPY_1_R-expressing cells

To prove NPY_1_R-mediated PPARγ activation by tesa-NPY, a PPARγ reporter gene assay in NPY_1_R-expressing HEK293 cells was performed. These assays demonstrated that tesa-NPY activated PPARγ, proving that it was successfully internalized and released. The specific receptor-mediated internalization of the peptide conjugate was confirmed by testing cells lacking NPY_1_R and by competition experiments using an excess of unlabeled NPY. Furthermore, cells that do not express PPARγ did not respond to tesa-NPY. Thus, the potency of the conjugate is focused on cells expressing NPY_1_R, enhancing PPARγ and possibly also PPARα activities, which we did not assess in our cell culture experiments. PPARα also expressed in the adipose tissue has been shown to attenuate adiposity by promoting adipocyte differentiation and suppressing adipocyte hypertrophy [45]. Therefore, its additional activation by tesaglitazar shuttled to adipocytes would additionally enhance the beneficial effects. In addition to adipocytes, neurons and vascular smooth muscle cells are the only known other cell types that express both NPY_1_R and PPARγ and thus might also be targeted by the peptide conjugate. The transport of PPARγ agonists to the CNS is hindered by the low NPY peptide permeability of the blood–brain barrier [[34], [35], [36]]. However, it can be expected that uptake might also occur in the vascular smooth muscle cells. The activation of PPARγ in vascular smooth muscle cells resulted in improved inflammation, coronary insulin resistance, and upregulation of adiponectin receptor expression and might even improve the health of patients with T2D [84,85]. In addition to the co-expression of NPY_1_R and PPARα in the adipocytes, both proteins are mainly co-expressed in the kidney (https://www.proteinatlas.org). The Y1 receptor is primarily a renal vascular receptor [86], and PPARα acts as an antiatherogenic factor by modulating local and systemic inflammatory responses. PPARα ligands have beneficial effects on diabetic nephropathy and have been shown to be protective in chronic kidney diseases [87]. Thus, targeting of vascular smooth muscle cells in addition to adipocytes should not lead to side effects.

### 4.3 Tesa-NPY enhances adipogenesis and is a suitable candidate for in vivo studies

After the initial proof of concept studies, further investigations revealed that tesa-NPY enhanced adipogenesis in murine 3T3-L1 cells, demonstrating that it was successfully internalized and released in cells natively expressing NPY_1_R. To strengthen this finding, we examined the expression of the PPARγ-responsive genes adiponectin, Fabp4, and Cidec [[71], [72], [73], [74],[88], [89], [90], [91]]. Adiponectin may mediate the beneficial effects of PPARγ agonists for the treatment of diabetes, as it is an insulin-sensitizing adipokine that affects hepatic glucose output and decreases triglyceride content in the liver and muscle [92]. Cha et al. showed that tesa induces increased adiponectin mRNA expression and plasma levels in *db/db* mice, which improves insulin resistance [93]. Fabp4 is needed to transport fatty acids through the cytoplasm, and Cidec is involved in lipid droplet formation, so both regulate lipid metabolism and are thus highly expressed in mature adipocytes [72,88]. Tesa and tesa-NPY enhanced the expression of adiponectin and shifted it to earlier time points in the differentiation procedure. Fabp4 and Cidec were only significantly induced by tesa treatment at the time points studied. Because the peptides were added simultaneously with highly optimized differentiation medium, the effect of treatment with tesa or peptides was overlaid by additional enhancing factors. This was reflected by a very strong increase in the adiponectin, Fabp4, and Cidec expression between days 3 and 4 even in the control DMSO treatment (for example, > 100 fold for adiponectin on day 4/day 3 and even higher for the other genes; data not shown). Furthermore, it was also underlined by the shift in the observed effects to earlier time points, which especially for tesa-NPY were hardly detectable on day 4 as opposed to day 3. The effects were easier to detect for tesa taken up by passive diffusion in substantial amounts than tesa-NPY, which was dependent on the receptor internalization. This was observed in the previously discussed PPAR reporter assays. To obtain more significant results, it would be necessary to optimize the relationship of the peptide treatment duration, enhancing the effect and minimizing the side effects in the differentiation protocol, already optimized to reach high differentiation rates in vitro. Nevertheless, significant results were obtained for 3T3 differentiation and the induction of adiponectin expression on day 3 and a similar trend was also observed in the SGBS cells, although it did not reach significant differences for tesa-NPY. These results illustrate that tesa-NPY can induce established PPARγ effects, such as induction of adipogenesis and adiponectin expression, which are beneficial for the treatment of diabetes as they lead to insulin sensitization. Thus, tesa-NPY possessed optimal requirements for in vivo studies.

### 4.4 In vivo studies demonstrate the anti-diabetic activity of the peptide-drug conjugate

Previous in vivo studies demonstrated that free systemically administered tesa can reduce hypertriglyceridemia, hyperinsulinemia, and hyperglycemia in *ob/ob* mice and restore insulin sensitivity in obese Zucker rats [43]. It also reduces atherosclerosis in LDL receptor-deficient mice [94]. In the present study, we used *db/db* mice to evaluate the anti-diabetic potency of the peptide conjugate, as these mice are known to develop severe insulin resistance and hypertriglyceridemia [95,96]. Phenotypical characterization revealed that the animals that did not receive tesa or tesa-NPY lost 3% of their body weight during the treatment period. During this time, all of the mice were 12–16 weeks old; *db/db* mice are known to suffer from severe disease syndromes such as progressive hyperglycemia and glucosuria, which lead to the loss of calories and thus body weight [[97], [98], [99]]. Treatment with tesa and tesa-NPY can prevent this weight loss, indicating a prevention of calorie loss through glucosuria by improved glycemic control as previously shown for other PPARγ agonists in diabetic ZDF rats [97,100]. Ljung et al. showed that tesa treatment does not lead to weight gain in treated animals, which is usually seen in traditional PPARγ agonists such as rosiglitazone [74]. In the present study, no body weight gain was detected and no major differences between the treated and untreated animals regarding body composition could be measured, as all of the groups lost fat and gained lean mass.

In contradiction to previous tesa in vivo studies, no major improvements in hyperglycemia (serum HbA1C) and serum insulin levels were detected in the treated mice compared to the untreated animals [43]. However, treatment with tesa or tesa-NPY did result in a deceleration of disease progression, which is characterized by increasing blood glucose values that could have been prevented by tesa and tesa-NPY in the present study. No major changes in the total serum glucose occurred and the insulin levels remained unchanged as the mice adapted to the amount of glucose in their blood. An additional reason for the constant insulin values might be the age of the animals, which was not comparable between the study of Ljung et al. who used 6-week-old mice, and the present study, which utilized 12- to 16-week-old mice [43]. *Ob/ob* and *db/db* mice up to three months old are characterized by strongly elevated serum insulin levels that are supposed to compensate for rising blood sugar concentrations. Afterward, disease progression results in a decline in serum insulin and decreased levels due to the destruction of pancreatic islets and the loss of β-cells [98]. At this stage, the destruction of β-cells might be too advanced to be restored by tesa treatment for only one week. Longer treatment periods might lead to better control of the glucose and insulin levels, which was previously shown for the PPARα/γ agonist muraglitazar in 12-week-old *db/db* mice, whereas treatment for one week did not lead to significant effects, whereas treatment for 2–4 weeks did [100].

Metabolomic studies of mice and humans have identified many plasma metabolite signatures in addition to HbA1C and insulin that are associated with the development and progression of T2D, such as high serum concentrations of FFAs, triglycerides, ketone bodies, leptin, and pro-inflammatory cytokines such as Mcp-1 and a low serum concentration of adiponectin [[101], [102], [103], [104], [105], [106], [107]]. All of these factors promote the manifestation of T2D. Increased concentrations of circulating FFAs, for example, are major factors eliciting systemic insulin resistance [108]. High plasma concentrations of FFAs, triglycerides, and ketone bodies and low levels of circulating adiponectin were normalized by tesa and tesa-NPY treatment, but no significant effect on leptin and Mcp-1 was detected. In summary, the tesa-NPY conjugate revealed promising anti-diabetic effects, as the present study demonstrated that it improved most of the aforementioned metabolic parameters and corrected hypertriglyceridemia.

As tesa-NPY, selectively transported to NPY_1_R-expressing cells, performed as well as systemically administered tesa, adipocyte-specific PPARγ activation is obviously sufficient for reversing metabolic parameters to a similar degree as systemic PPARγ activation. This was demonstrated by Sugii et al. who used PPARγ agonists to restore the whole body insulin resistance of HFD mice that expressed PPARγ in an adipocyte-specific manner as efficiently as mice that expressed PPARγ systemically [109]. Thus, the observed anti-diabetic effects are assumed to result from adipocyte-specific PPARγ-mediated mechanisms. These include the release of insulin-sensitizing and anti-inflammatory adipokines such as adiponectin, and the lowering of plasma FFA and TG levels by uptake into newly differentiated adipocytes [9,110]. Both these effects were detected in the present study. Adipose tissue histology confirmed smaller adipocyte size and thus adipogenesis only in the mice treated with tesa-NPY. Tesaglitazar is an orally active agent on a whole body level [43], whereas tesa-NPY acts selectively on NPY-expressing tissues such as adipose tissue and must be administered intraperitoneally. Both tesa and tesa-NPY improve the metabolic state but tesa-NPY selectively acts on adipose tissue. Studies of volunteers demonstrated that tesaglitazar is rapidly and completely absorbed after oral dosing and has linear pharmacokinetic properties, with an elimination half-life of between 38 and 59 h [111]. Therefore, only tesa-NPY can exert marked effects on adipose tissue. In agreement with this phenotype, the KEGG pathways showed increased fatty acid transport through Fabp4, although the LPL pathway was downregulated. Furthermore, perilipin decreased blunting access to the TGs and therefore reduced sequestration by active lipases. The fatty acid and beta oxidation pathways were significantly enriched. These mechanisms warrant the potential of tesa-NPY for metabolic disease improvement.

In line with the morphological changes in the adipose tissue and metabolism under tesa-NPY, the microarray data analyses demonstrated differential increases and decreases in the genes highly relevant to glucose metabolism and adipogenesis. The loss of *RhoB*, a downregulated gene in animals treated with tesa-NPY compared to [F^7^, P^34^]-NPY, has been shown to prevent streptozotocin-induced diabetes and ameliorate diabetic complications in mice, which could indicate the positive effects of tesa-NPY in the absence of HbA1c differences between the delay in disease progression in animals under treatment [112]. *Lims2* was downregulated in tesa-NPY treatment. In the literature, *lims2* is described as interacting with *GP17*, whose deletion has been shown to increase body weight without changing food intake although lims2 deficient mice do not demonstrate a specific obese phenotype [113]. The downregulation of *Lims2* under tesa-NPY can mediate the prevention of weight loss in diabetic animals compared to untreated animals. In addition, *FGFRL1* decreased under tesa-NPY. *FGFRL1* is the fifth member of the fibroblast growth factor receptor (*FGFR*). It interacts with fibroblast growth factors to induce differentiation and plays a key role during embryonic development [114]. This gene is highly expressed in subcutaneous and epigonadal fat pads in mice. The expression of *FGFRL1*and *FGFR1* increased during adipocyte differentiation from mesenchymal stromal cells, was more highly expressed in preadipocytes compared to adipocytes, and *FGFR1* knockdown further inhibited adipocyte differentiation [115]. However, mice treated with tesa-NPY demonstrated unregulated *CXCL13*, which has been shown to be highly expressed in mature adipocytes compared to preadipocytes and mediate B-cell uptake to the liver in *ob/ob* mice [116]. The interplay of these genes in adipogenesis and differentiation could demonstrate the essential role of tesa-NPY treatment in active adipocyte differentiation and maturation, supporting data from the AT histology and cell culture studies presented.

The mice treated with free tesa showed a trend toward smaller adipocyte size and also demonstrated a reduction in plasma FFAs and TGs. This was because the insulin-sensitizing effects of PPARγ agonists do not depend only on adipogenesis. Sugii et al. showed that PPARγ activation in mature adipocytes but not preadipocytes (and hence no activation of adipogenesis) was sufficient to improve insulin sensitivity [109]. Thus, lower plasma FFA and TG levels can be explained by the second PPARγ-mediated mechanism, which is the release of adipokines such as adiponectin. Adiponectin is known to enhance FFA uptake and oxidation in the muscle and liver, leading to decreased circulating FFAs [[92], [117]]. The reduction in plasma FFAs then correlates with a mobilization of lipids out of the liver and muscle, thus improving whole body insulin sensitivity [[110], [118]].

Impaired thermoregulation is another well-documented characteristic of diabetic mice. At temperatures of approximately 30 °C, which were used in the present study, the body temperature of *db/db* mice was approximately 1 °C lower than that of lean C57BL/6N mice [119,120]. The body temperature could be normalized by treatment with tesa, tesa-NPY, and [F^7^, P^34^]-NPY, proving that not only tesa but also [F^7^, P^34^]-NPY can have beneficial effects. This is in accordance with the literature, as the body temperature of mice increases if they are administered NPY in the paraventricular nucleus, yet no investigation in mice has been performed to date demonstrating the same effect for NPY administered peripherally [121]. Moreover, in endotoxemic rats, a single dose of peripherally injected NPY stabilized their body temperature [122].

In contrast to adipose tissue, there is very low expression of PPARγ and even less NPY_1_R in human and mouse livers (http://www.informatics.jax.org/expression.shtml; https://www.proteinatlas.org). We found that tesaglitazar significantly enhanced the expression of PPARγ in the liver. Tesa is a dual agonist of PPARα and γ that improves both lipidemic and glycemic abnormalities in preclinical models of type 2 diabetes and metabolic syndrome [43, 123,124]. The tesa-NPY conjugate also elevates PPARγ expression but not significantly. That elevation might be due to the fatty liver in *db/db* mice. *Db/db* mice exhibit non-alcoholic fatty liver disease (NAFLD) with adipocytes. Further investigations will be required to determine whether isolated hepatocytes represent stable noninfluenced PPARγ expression in non-fatty tissues.

## 5 Conclusion

In conclusion, we developed a novel system for the cell-type specific uptake of a PPARα/γ dual agonists by peptide-mediated internalization and controlled release into adipocytes. Treatment of adipocytes with peptide conjugate enhanced adipogenesis and adiponectin expression. In vivo studies using *db/db* mice proved the ability of the peptide conjugate to prevent diabetes progression by reducing plasma FFAs and hypertriglyceridemia and enhancing plasma adiponectin levels as efficient as systemically administered tesa. These data clearly demonstrated that a peptide conjugate composed of [F^7^, P^34^]-NPY and tesa is as efficient as free tesa and is thus a promising drug candidate that potentially reduces the known side effects of non-selective PPARγ agonists for the treatment of T2D.

## Author contributions

SW and AB-S conceived the study, designed and conducted the experiments, analyzed the data, and wrote the paper. NK conducted the mouse experiments, contributed to the discussion, and reviewed the manuscript. RC performed the microarray data analyses, wrote respective sections, contributed to the discussion, and reviewed the manuscript. KM and MB reviewed the manuscript. All the authors discussed the results and commented on the manuscript. AB-S supervised the project.
