# Supplementary material for: NPY1R-targeted peptide-mediated delivery of a dual PPARα/γ agonist to adipocytes enhances adipogenesis and prevents diabetes progression
Source: Mol Metab. 2019 Nov 16;31:163–80. doi: 10.1016/j.molmet.2019.11.009 (PMC6931124; doi:10.1016/j.molmet.2019.11.009)
Supplement: Multimedia component 1 [file mmc1.docx]

**Table S1: TesaNPY_vs_FP_NPY_upregulated genes**

| logFC "AveExpr" "t" "P.Value" "adj.P.Val" "B" |
| --- |
| Cyp2f2 5.44246488977213 8.76760359881106 4.25857721400177 0.00522504037346718 0.721037399657979 -3.90247758967388 |
| Cyp2e1 4.60414264702316 13.9978667840944 4.72775488687481 0.00315782228360406 0.721037399657979 -3.85365792388808 |
| Ighv3-6 4.05395011597839 8.34857169882455 4.20421464064088 0.00555051017623956 0.721037399657979 -3.90880488314574 |
| Igkv3-10 3.72973354625719 8.1675986104829 6.22676289761401 0.000767088783565659 0.721037399657979 -3.74879954372969 |
| Ighv1-4 3.12386440330573 7.36220648584986 3.93930160811689 0.00749863628473075 0.721037399657979 -3.9418896542102 |
| Cxcl13 3.0089803961951 11.4877038916033 6.56676095927459 0.000576682551043659 0.721037399657979 -3.73247689667145 |
| Ces1d 2.94653992274302 9.65037377949216 4.49036554002748 0.00405807821796683 0.721037399657979 -3.87712063009044 |
| Ighv2-9-1 2.41433957059453 8.96629973244345 7.74536846901382 0.000233170770603559 0.721037399657979 -3.68914960310173 |
| Ighv2-6-8 2.33956376763721 8.93674232563921 8.47734674831746 0.000140573689764323 0.721037399657979 -3.66972811966527 |
| Nrn1 2.19970068845879 8.84648913860944 4.82890624886673 0.00284455988812704 0.721037399657979 -3.84436357949436 |
| Krt18 2.19123747106521 6.95110611964004 3.75500041946097 0.00930287247898771 0.721037399657979 -3.96728413303823 |
| Igkv4-68 2.14847607778499 6.31840630753425 3.94467403681663 0.00745223842397658 0.721037399657979 -3.94117977903825 |
| Igkv3-2 2.01258062680686 7.40452990474135 3.70006275332934 0.00993041380517895 0.721037399657979 -3.97525965071382 |
| Ighv9-1 2.0096042186157 7.72825973767356 3.98556107485104 0.00710943202310073 0.721037399657979 -3.93583177338086 |
| Dpyd 1.86358232800928 8.8236901858457 4.28808114208349 0.00505739301529754 0.721037399657979 -3.89910600646836 |
| Krt8 1.76104241907466 7.53010659146079 4.28606962354564 0.00506862924262104 0.721037399657979 -3.89933449738773 |
| Ighv8-8 1.75463956935698 7.35293081946586 4.07085017170392 0.00644954027795463 0.721037399657979 -3.92498023186395 |
| Ighv9-4 1.73379109244139 6.9558228188036 4.3936840982675 0.00450496053246565 0.721037399657979 -3.88738746847959 |
| F830016B08Rik 1.59541654373476 8.07870082418404 3.70889453308231 0.00982643819910017 0.721037399657979 -3.97396452631378 |
| Nipsnap1 1.45613067574814 7.39363135782086 3.7213133893712 0.00968226835886145 0.721037399657979 -3.97215183133965 |
| Gm24669 1.3739309870275 6.8438999119711 7.5354926970537 0.000271598755444575 0.721037399657979 -3.69563003175954 |
| Gm22488 1.36627807753013 8.10751440235051 4.19812940004149 0.00558833512284081 0.721037399657979 -3.90952255424466 |
| Erbb3 1.32300980151012 6.85258338878703 3.71029848172932 0.00981002110770978 0.721037399657979 -3.97375910705441 |
| Gm24149 1.27431170049121 11.444134446412 4.85439217428114 0.00277127465406623 0.721037399657979 -3.84208423832367 |
| Gm25618 1.2718247537064 12.0041569645836 3.91498738175869 0.00771268533435515 0.721037399657979 -3.94512338968996 |
| Mir1946b 1.24758809309545 10.8165460314956 4.04347700128377 0.00665356580919762 0.721037399657979 -3.92841882639717 |
| Igkv3-4 1.24296486261246 6.05747491093152 5.29508479576313 0.00178904014393155 0.721037399657979 -3.8063000303894 |
| Gm4788 1.24013243193279 9.88070667503537 5.13572612769852 0.00208964261928159 0.721037399657979 -3.81848679205864 |
| Gm24592 1.23489464502379 12.5533172891469 4.03139037015765 0.0067459394879034 0.721037399657979 -3.92995033302863 |
| Gm26122 1.23010712781966 11.9742322279638 3.76815762690154 0.00915919573840964 0.721037399657979 -3.96540237682492 |
| Akr1c19 1.22959876352642 6.17281297215446 3.8664142728408 0.00816097750088364 0.721037399657979 -3.9516878033749 |
| Gm25812 1.22680122762929 8.14965391966079 4.29989493979213 0.00499196310228588 0.721037399657979 -3.89776810260065 |
| Ighv8-6 1.22106252646806 6.96346243618679 3.81024277202136 0.00871591818043197 0.721037399657979 -3.95945570907405 |
| Dsg2 1.21484889333179 6.10754339872475 4.35314932714229 0.00470857265256735 0.721037399657979 -3.89182190583816 |
| Id2 1.19711974320037 11.8340976991482 5.85403834016811 0.00106359603916982 0.721037399657979 -3.76929878516299 |
| Aspg 1.18389730824 5.75652770543996 3.91561999386695 0.00770703065932413 0.721037399657979 -3.94503881554549 |
| Igkv3-3 1.16495295466985 6.33852218079986 4.21572614415994 0.00547973818295856 0.721037399657979 -3.90745247264028 |
| Aldh7a1 1.15979508737107 9.50639120876853 3.69842963691369 0.00994977360246232 0.721037399657979 -3.97549968587653 |
| Gm23138 1.15328105237119 9.11563052997356 4.13959737274822 0.00596727714961914 0.721037399657979 -3.91652411743204 |
| Gm26430 1.14461659954712 12.7089876319209 3.819374227748 0.00862291694530079 0.721037399657979 -3.95817985742769 |
| Gm5834 1.13283381461663 10.0496357649646 4.1023201035876 0.00622357077318601 0.721037399657979 -3.92107769009524 |
| Gm23846 1.12160808966521 8.58900386984249 5.00048299266932 0.00239023280735117 0.721037399657979 -3.82948256757145 |
| Gm22308 1.11199026961277 13.2605319174302 3.78740724375635 0.00895340848944392 0.721037399657979 -3.96266876427142 |
| Gm26071 1.09201519275866 10.7210853163872 3.92268016342153 0.0076442349321757 0.721037399657979 -3.94409653140474 |
| Gm25538 1.08582480411751 7.42174559877693 5.44016448059788 0.0015574885143627 0.721037399657979 -3.79587938765406 |
| Ighv6-7 1.0842504601939 6.81960992118979 5.28728142364558 0.00180256215156815 0.721037399657979 -3.8068782839738 |
| Gm23412 1.08142843460559 12.6606209621705 3.74903141384454 0.00936887747619094 0.721037399657979 -3.96814141104041 |
| Gm22392 1.07321943846495 12.9863097957897 4.20989029887602 0.00551548943873012 0.721037399657979 -3.90813723649437 |
| Gm19980 1.07078272414564 7.41902025007083 3.74286049417073 0.00943766207582753 0.721037399657979 -3.96903004912804 |
| Gm26485 1.05662524396853 10.4068602568338 3.97102358373539 0.00722926118148368 0.721037399657979 -3.93772228010867 |
| Gm25810 1.05153050336352 11.4137727512507 3.80627725242127 0.00875665257330049 0.721037399657979 -3.96001136745341 |
| Gm25815 1.01852918663055 9.10986395669791 4.69821216212232 0.00325653549988156 0.721037399657979 -3.85644909255991 |
| Gm26228 1.01395262074337 7.48740688426536 5.04225509904393 0.00229245295697265 0.721037399657979 -3.82601965651609 |
| Cfhr2 1.01302303679701 9.98960317474951 3.83048605084449 0.00851123090292908 0.721037399657979 -3.9566341877496 |
| Gm23151 1.00300305734691 10.418850977668 3.73919997321633 0.00947872900852665 0.721037399657979 -3.96955831598624 |
| Gm24121 0.997627506064211 8.58738411834368 6.35423529012504 0.0006883171130391 0.721037399657979 -3.74243455302526 |
| Gm24457 0.992922689149538 7.61833788591339 4.46434579840014 0.00417323636274088 0.721037399657979 -3.87984152603714 |
| Hook1 0.991956690379319 6.45012205578964 3.88439030956741 0.0079917764516869 0.721037399657979 -3.949242097914 |
| Gm24917 0.978813278790377 7.98429531816394 4.02271070111808 0.00681315839303738 0.721037399657979 -3.93105516333349 |
| Gm25897 0.976204194522512 8.20094896391817 4.81084477056522 0.00289782257668563 0.721037399657979 -3.84599392616898 |
| Gm22753 0.972491916184983 7.54197515940669 4.3094424343733 0.00493977847224689 0.721037399657979 -3.89669188249317 |
| Gm25581 0.964353856553877 12.7807951729293 3.83718467035778 0.00844467992646541 0.721037399657979 -3.95570603575702 |
| Gm25354 0.940615307745073 8.01674316195936 5.54645472503147 0.00140941515037969 0.721037399657979 -3.78862632155849 |
| Gm22045 0.937441122009543 8.84950333898889 3.73074622228347 0.0095743303300267 0.721037399657979 -3.97078155887891 |
| Gm25020 0.934750234529633 9.1520913103132 6.44766705174661 0.000636442704746055 0.721037399657979 -3.73795958332085 |
| Tnfrsf13c 0.932156061680903 5.50007339423333 3.84475591138752 0.00837015516993336 0.721037399657979 -3.95466025443065 |
| Gm25039 0.931487656244584 11.1376773563035 3.8639527952656 0.00818445513167364 0.721037399657979 -3.95202420138119 |
| Gm23540 0.910214492167759 7.67146839588826 3.83714333932578 0.00844508877322741 0.721037399657979 -3.95571175416863 |
| Gm25818 0.90687489171225 15.1253397717957 4.21454882068264 0.00548692968605878 0.721037399657979 -3.9075904762078 |
| Gm22151 0.903042005377777 8.01377736309929 4.79225518540069 0.00295382268841919 0.721037399657979 -3.8476850800104 |
| Gm22113 0.898730068542636 9.81772711940505 3.81492381545112 0.00866810459674881 0.721037399657979 -3.95880103271795 |
| Gm11192 0.897569137107823 7.43408987584318 3.71587152395823 0.00974515195994889 0.721037399657979 -3.97294492972055 |
| Gm22454 0.893051512105301 6.41417163419909 4.28015812940943 0.00510181290606586 0.721037399657979 -3.90000715361486 |
| Gm23928 0.887200985329029 14.3662075030889 4.01924063334095 0.00684024097448929 0.721037399657979 -3.93149804543239 |
| Gm24842 0.88396803073567 11.2329191920295 4.55215684776356 0.00379866032793482 0.721037399657979 -3.87078052889708 |
| Gm23730 0.86920076564232 10.2340406188842 4.09429744725921 0.00628032493639621 0.721037399657979 -3.92206745917641 |
| Gm23096 0.864019077040578 7.29503070202314 3.69569272247725 0.00998231225417718 0.721037399657979 -3.97590234198609 |
| Gm22297 0.863830874384189 6.46270702633754 5.05760034172809 0.00225767738245718 0.721037399657979 -3.82476269386506 |
| Tmem102 0.855515947108224 5.36870169691492 3.88681270943132 0.00796927543387089 0.721037399657979 -3.94891399496274 |
| Gm8260 0.853460476915585 11.3754361007159 4.8713167752196 0.00272378746237728 0.721037399657979 -3.84058414864188 |
| Mpzl2 0.846949244458905 5.77190875959947 3.71008492444462 0.00981251638038822 0.721037399657979 -3.97379034562442 |
| Gm23378 0.84543625825168 5.97030620607362 3.77293648484992 0.00910762187404328 0.721037399657979 -3.96472158285038 |
| Gm26245 0.842051195846651 9.03205937027275 3.79291642875006 0.00889546174317521 0.721037399657979 -3.9618906503308 |
| Gm23097 0.8228756560282 5.7886443055268 5.06919302899136 0.00223180209637545 0.721037399657979 -3.82381845224349 |
| Gm25134 0.822175509354755 9.71043023200785 4.05469689919577 0.00656907904734391 0.721037399657979 -3.9270043959899 |
| Gm5611 0.81241257419891 8.02183506999108 4.36241843699121 0.00466111655904752 0.721037399657979 -3.89080097156141 |
| Trav3-1 0.808791554911 6.10429963935753 6.24252933983131 0.000756807532859563 0.721037399657979 -3.74799552623453 |
| Gm22260 0.808056432272346 9.64409572694021 4.49259006307348 0.00404840001247091 0.721037399657979 -3.87688943037074 |
| Gm24705 0.800782528004912 8.84204510695473 4.52227248745982 0.00392171592405722 0.721037399657979 -3.87382567108545 |
| Gm23749 0.793013193425099 6.74068477500442 4.1373801810578 0.00598218645271189 0.721037399657979 -3.91679288083151 |
| Snord90 0.784410427673327 6.82990150679895 5.22071385758502 0.00192275192436562 0.721037399657979 -3.81188751245583 |
| Gm22126 0.784181527238353 14.8560778762049 3.7934895814845 0.00888945716882262 0.721037399657979 -3.96180980651916 |
| Gm24316 0.783034837238173 6.02974196712807 4.00450793418543 0.00695656999867583 0.721037399657979 -3.93338589869914 |
| Gm23987 0.782716073016125 6.47197771267358 4.93212204601456 0.0025606158617151 0.721037399657979 -3.83528260430245 |
| Igsf5 0.78049519092874 5.61568042303344 3.92282181684141 0.00764298086133431 0.721037399657979 -3.94407765553732 |
| Gm15538 0.773405757123143 10.1348073240612 4.24718128771816 0.00529145534941289 0.721037399657979 -3.90379154254935 |
| Gm25204 0.7706272861435 6.24888321166639 4.52928954163534 0.00389242339305323 0.721037399657979 -3.87310711452728 |
| Igkv1-99 0.762362541565413 6.29784187931946 4.22419299363055 0.00542832967704334 0.721037399657979 -3.90646209513326 |
| Gm11913 0.754571937069871 5.53989503035206 3.73108700189066 0.00957045591515365 0.721037399657979 -3.97073216098669 |
| Gm22717 0.750168730364278 6.09970317458195 4.28954160348487 0.00504925246860214 0.721037399657979 -3.89894023645814 |
| Gm26442 0.741765176691681 10.2137666315608 4.08708760729561 0.00633182299844274 0.721037399657979 -3.92295992524217 |
| Gm15199 0.739612811124212 6.17242266809545 4.8006004094848 0.00292853328556725 0.721037399657979 -3.84692423240893 |
| Gm25329 0.739492601936106 4.75787144705384 4.97157501465461 0.00246066623839231 0.721037399657979 -3.83191487877242 |
| Gm25589 0.735430558606505 6.04635691934826 4.16727715214583 0.00578460476404021 0.721037399657979 -3.91319071102203 |
| Gm13313 0.734557949458777 6.59395913722643 4.92770382683075 0.00257209224908466 0.721037399657979 -3.83566324820868 |
| Olfr1198 0.733165771659918 5.54411819747882 6.39850043604619 0.000663160152138578 0.721037399657979 -3.74029491326124 |
| Gm6461 0.732440125777313 7.14948998656967 4.52317727798248 0.0039179249959364 0.721037399657979 -3.87373289677964 |
| Gm25986 0.730686070851016 9.25589788624259 5.26904401380324 0.00183461974370112 0.721037399657979 -3.80823699996158 |
| Gm22977 0.724845564111344 7.07990184364709 4.14225524077114 0.00594945932032904 0.721037399657979 -3.91620228004664 |
| Gm23615 0.720026698526571 6.11129643407371 4.28736762106457 0.00506137550208226 0.721037399657979 -3.89918703326293 |
| Gm24415 0.719932564160687 6.60191625349808 5.6350065708398 0.00129821162800687 0.721037399657979 -3.7828168011517 |
| Gm22155 0.716131363100488 14.4321751160768 4.0587668277622 0.00653872887423686 0.721037399657979 -3.92649304124706 |
| Gm12431 0.713449244694645 6.27779549268267 3.94320521935941 0.00746489169854488 0.721037399657979 -3.94137369199802 |
| Gm23435 0.70996981870613 7.11261374181509 3.70962170139194 0.00981793124788165 0.721037399657979 -3.97385811465359 |
| Gm5942 0.706219046114293 6.24512847689636 4.06939553817754 0.00646020479601917 0.721037399657979 -3.92516192464075 |
| Gm9060 0.704540217553195 5.69410266200291 4.15301187444968 0.00587795443451278 0.721037399657979 -3.91490359534961 |
| Gm24626 0.690306056062486 7.11563895508935 4.30443358543971 0.00496707906899035 0.721037399657979 -3.89725593498861 |
| Mir107 0.682635954750076 6.27692313434613 4.90523978715244 0.00263135065419102 0.721037399657979 -3.83760962827644 |
| Gm9522 0.680384184591793 8.06658980665425 4.21193133710887 0.00550295610636546 0.721037399657979 -3.90789754727064 |
| Gm19840 0.676137078605861 5.16919806527214 3.81792969555255 0.00863755536461645 0.721037399657979 -3.95838134797451 |
| Gm23678 0.674528670268954 10.351902255822 3.89497258479543 0.00789399789532347 0.721037399657979 -3.94781134143784 |
| Gm24735 0.673384144179182 6.58641974700644 4.17195219393187 0.00575437398253089 0.721037399657979 -3.91263168548435 |
| Gm14650 0.667921531907472 8.9547085630914 3.7225854817229 0.00966763364005204 0.721037399657979 -3.97196670879413 |
| Gm11749 0.662747017731717 5.0866622043731 4.35441978387041 0.00470203618031941 0.721037399657979 -3.89168172924152 |
| Gm23462 0.662413795561183 7.84230328503209 3.80964136891922 0.00872208231169836 0.721037399657979 -3.95953991698025 |
| Gm23966 0.662063776611108 7.17507302872131 4.61713540375797 0.00354581910386753 0.721037399657979 -3.86429332095141 |
| Gbp11 0.649747036053681 8.14344009125274 5.29848594351564 0.00178318257319805 0.721037399657979 -3.80604857545873 |
| Gm12044 0.649557567908774 4.38887333873042 3.94006730784726 0.00749200384881268 0.721037399657979 -3.94178837763866 |
| Gm20513 0.648081115493775 6.1811782918754 5.65160457797593 0.00127849866368149 0.721037399657979 -3.78175066029023 |
| Olfr794 0.647442164095511 5.08609467412523 4.35188476628525 0.00471508892479296 0.721037399657979 -3.89196150893496 |
| Gm17604 0.641339064272653 10.2857617742536 5.73804669670009 0.00118118917590752 0.721037399657979 -3.77631097227612 |
| Gm23470 0.640803059848027 6.96250815953881 5.17620353287708 0.00200819007133512 0.721037399657979 -3.81531468693272 |
| Gm25526 0.640380082259506 6.87602648839655 3.89799491116021 0.00786631713250978 0.721037399657979 -3.94740393253782 |
| Gm14275 0.633170613810492 6.98722149571766 4.22040475678985 0.00545126376416711 0.721037399657979 -3.90690475647008 |
| Pramef17 0.627060599854639 4.96809883083052 5.53461936100415 0.00142508399965396 0.721037399657979 -3.78941859793961 |
| Gm22264 0.626846886160602 7.96496028804031 3.89971350142524 0.00785062518814435 0.721037399657979 -3.94717250798283 |
| Gm24921 0.622703471932276 6.13272307897161 4.16048139556484 0.00582886652048812 0.721037399657979 -3.91400536792124 |
| Gm11111 0.619890777996223 7.98027669557843 4.76409020161147 0.00304101322338716 0.721037399657979 -3.85027299708843 |
| Gm11172 0.61897009398925 10.9721689260224 3.85317629775559 0.00828813008921749 0.721037399657979 -3.95350125923252 |
| Gm25343 0.618050625278023 5.88386719271718 4.54970141080369 0.00380860549177395 0.721037399657979 -3.87102925245698 |
| Gm22739 0.602364586882053 13.333752816395 3.72808442464906 0.00960465295920265 0.721037399657979 -3.97116765505347 |
| Gm12954 0.60203350863916 4.7816016661694 5.77173176051881 0.00114557675858731 0.721037399657979 -3.77424132840687 |
| Olfr44 0.594875179979597 6.66369720481853 3.97590140810222 0.00718880510763394 0.721037399657979 -3.93708660493871 |
| Olfr1123 0.591014193974348 5.15596765827088 4.70469344883222 0.00323458440370041 0.721037399657979 -3.85583372065782 |
| Mir7213 0.587135499459353 6.62502567348421 3.76136425809634 0.00923306934088434 0.721037399657979 -3.9663726155205 |
| Gm16158 0.58702426107396 5.12595947968864 3.85087182975288 0.00831048926610733 0.721037399657979 -3.95381802417201 |
| Gm15994 0.585967343554894 4.88208833279092 3.75407676867547 0.00931305233127093 0.721037399657979 -3.96741664258856 |
| Vmn1r234 0.58389412985728 5.3729864406173 4.08440433922194 0.00635110928598206 0.721037399657979 -3.92329279328826 |
| Gm25677 0.580447071304652 5.60178627255059 3.73174574656802 0.00956297139926872 0.721037399657979 -3.97063669319911 |
| Gm12196 0.580045129933511 7.45531534966224 3.86714908380942 0.00815398338826078 0.721037399657979 -3.95158745082046 |
| Gm20305 0.577017333910488 10.1997076064661 3.80710671487686 0.00874811474827888 0.721037399657979 -3.95989506115741 |
| CLRN1-AS1 0.575035624773418 4.99999981098785 4.55742567254134 0.00377741876381448 0.721037399657979 -3.87024771205707 |
| Gm24218 0.568733316343457 4.99343889712966 3.92142563301634 0.0076553514167257 0.721037399657979 -3.94426375370115 |
| Gm13714 0.565915201985363 6.47911709645877 3.94570583590948 0.00744336418237411 0.721037399657979 -3.94104363591629 |
| Olfr1271 0.559313339116792 5.05330757131383 4.37266374889453 0.00460928643929867 0.721037399657979 -3.88967729688 |
| Gm15778 0.558833085264486 8.18992436136679 3.85085078072223 0.00831069380400599 0.721037399657979 -3.95382091898276 |
| Gm15785 0.556651732669997 12.0779226860834 4.2476394070953 0.00528876726326708 0.721037399657979 -3.90373859497529 |
| Gm20755 0.554759254116792 4.99976651495273 4.10410245281228 0.00621104000257076 0.721037399657979 -3.92085827083754 |
| Mir139 0.554603025977156 8.60974308419346 3.94344034491256 0.00746286457722275 0.721037399657979 -3.94134264236516 |
| 4930529L06Rik 0.554295606539265 6.01128655832718 4.58833069628758 0.00365548536179485 0.721037399657979 -3.86714666861583 |
| Ccr7 0.553816150715669 7.14117249230714 3.9220095256559 0.0076501752630032 0.721037399657979 -3.944185912427 |
| Gm24200 0.55075576479418 5.89080260751819 4.34342626368869 0.00475893680278983 0.721037399657979 -3.89289727755251 |
| Gm16308 0.547548423301294 4.43908490207078 4.60550224812741 0.00358965661168598 0.721037399657979 -3.86544142430326 |
| Gm14417 0.546870245116017 7.96637677149734 4.18175822933615 0.0056915369003514 0.721037399657979 -3.91146282366424 |
| Gm24686 0.546831206145656 5.39245284499691 4.56569265703672 0.00374435904328101 0.721037399657979 -3.86941414024512 |
| Vmn2r-ps71 0.545790608662896 5.47718029289332 3.81764047090086 0.00864048959080714 0.721037399657979 -3.95842170581796 |
| Gm17187 0.541492637906153 5.92204529467646 3.99178721190554 0.00705879040331897 0.721037399657979 -3.93502578786432 |
| Ccdc184 0.534950839973033 6.55192087757222 4.17998037696838 0.00570287213385851 0.721037399657979 -3.9116743682615 |
| Mir465b-1 0.533805771195577 7.80396979633215 4.21852169166825 0.00546270427101994 0.721037399657979 -3.90712506853528 |
| Gm10283 0.533150710137109 5.78259738477446 3.9838261279933 0.00712361569634332 0.721037399657979 -3.93605675840115 |
| Vmn2r-ps80 0.529530895296031 6.55418468122389 4.339133666024 0.00478136411142421 0.721037399657979 -3.89337349024558 |
| Gm5228 0.525518814249513 6.31263623573867 4.50013528201262 0.00401576606407923 0.721037399657979 -3.87610689477273 |
| Gm22741 0.524910648975175 5.59774874126865 4.47373165299213 0.00413127779383461 0.721037399657979 -3.87885650483175 |
| Gm22684 0.522225949726378 6.59269872487399 4.01644705896689 0.006862131104135 0.721037399657979 -3.9318550776455 |
| Gm14537 0.518031259262176 7.15150750858132 4.26596103698631 0.00518250687100164 0.721037399657979 -3.90162971859062 |
| Mir6965 0.516234809494008 6.54618697464634 3.86047948829362 0.00821771155321788 0.721037399657979 -3.95249949992642 |
| Olfr231 0.515318426742983 5.56513609282717 4.59099336860255 0.00364518921539721 0.721037399657979 -3.86688142134007 |
| Gm23586 0.513320790287498 6.05748014060901 4.0780569759398 0.00639699355962193 0.721037399657979 -3.92408176524478 |
| Gm26273 0.511096381260598 7.22956364705963 4.1315062517175 0.00602188700945001 0.721037399657979 -3.91750616986535 |
| Rpl23a-ps4 0.507661105304195 7.33386404865259 4.09982630839422 0.00624115081790507 0.721037399657979 -3.92138498110607 |
| Gm23014 0.507102325646287 8.26549802154575 4.47489576398219 0.00412610690637377 0.721037399657979 -3.87873461340688 |
| Prrg4 0.503049683817504 6.06218731361899 4.07866763346447 0.00639256312694447 0.721037399657979 -3.92400576549584 |
| Gm4405 0.499960413824952 7.83305869284403 4.23045779245411 0.00539063926574783 0.721037399657979 -3.90573165064849 |
| Gm13681 0.49246685944612 7.1958661537189 4.19421315024523 0.00561283060261101 0.721037399657979 -3.90998543406828 |
| Gm5899 0.49205995444542 7.75558082884254 4.02458955636308 0.00679854459694486 0.721037399657979 -3.93081564781898 |
| Gm17234 0.491904532139334 5.19213293000119 4.49186251527225 0.0040515624847303 0.721037399657979 -3.87696502156111 |
| 1700013D24Rik 0.487654069506158 6.17322146577454 3.84044670732071 0.00841248115580663 0.721037399657979 -3.9552550391863 |
| Olfr119 0.487401403839758 5.2729717091752 5.23521138510076 0.00189582020769998 0.721037399657979 -3.81078481057221 |
| Gm17753 0.485804052279693 5.08165516379611 3.9029903308283 0.00782080191615277 0.721037399657979 -3.94673173533458 |
| Fam169b 0.485473999261373 5.49187071036647 3.70982318380512 0.00981557560281854 0.721037399657979 -3.97382863630652 |
| Mir721 0.485130303199002 8.34210041587933 3.81467797056153 0.00867060846465574 0.721037399657979 -3.95883538238359 |
| Gm11763 0.476560569429829 5.65333065820827 3.78682715455513 0.00895953434629345 0.721037399657979 -3.96275080518323 |
| Rpl31-ps5 0.473079282240885 5.78615701667671 3.76887182096726 0.00915146752482805 0.721037399657979 -3.96530054237123 |
| Gm19601 0.468924808808179 8.94395321716014 4.17829489336768 0.00571364178728058 0.721037399657979 -3.91187507408695 |
| Gm9 0.448971939237792 5.22817839878569 3.96539694627983 0.00727624348689654 0.721037399657979 -3.93845723317321 |
| Stmn1-rs1 0.442754713768249 6.54547868125588 4.39001836736455 0.00452296131798567 0.721037399657979 -3.88778528542445 |
| Gm23100 0.440607427973605 6.37808013198119 3.9135579179541 0.00772547978561285 0.721037399657979 -3.94531458177422 |
| Gm24665 0.438191884486202 6.47727303591035 3.85710656377183 0.00825015077599762 0.721037399657979 -3.95296175594724 |
| Gm23221 0.434297745333576 6.46700250773103 3.98351319950286 0.00712617733243672 0.721037399657979 -3.93609735669328 |
| Tert 0.42819174415698 5.19542547179895 3.73438562453335 0.00953304275007833 0.721037399657979 -3.97025438834438 |
| Gm14110 0.427811938263253 5.72219321483068 3.92339063008729 0.00763794741258121 0.721037399657979 -3.94400187078937 |
| Magea5 0.42688072421085 5.44415730523851 3.82468662484421 0.00856931954682035 0.721037399657979 -3.95743995540501 |
| Mrgpra3 0.425545638449049 6.26413247976571 3.92394617471347 0.00763303494290805 0.721037399657979 -3.94392787213063 |
| Gm25301 0.424672934871056 4.49026713887822 3.96129463706726 0.00731071229505881 0.721037399657979 -3.93899422372615 |
| Gm15729 0.423437634458721 5.93897287131538 4.4636713262276 0.00417626993852378 0.721037399657979 -3.87991246458277 |
| Gm24166 0.419578747958157 5.70616696386155 3.87484523039061 0.00808112897543563 0.721037399657979 -3.95053833708152 |
| Gm16330 0.418096705024343 4.64152917129206 3.96472143439869 0.00728190685235071 0.721037399657979 -3.93854559088249 |
| Gm13383 0.417550348660685 6.07397912171367 4.30079935551624 0.00498699332414799 0.721037399657979 -3.89766596207039 |
| Ighv2-1 0.408160235838061 7.06030224192303 4.1219727077185 0.00608695099923408 0.721037399657979 -3.91866777237722 |
| Ssxa1 0.407515181681073 4.80302744247146 3.96956062239079 0.00724144424967116 0.721037399657979 -3.93791319791155 |
| Gm16481 0.396141298815724 4.39634965822047 3.77295913184031 0.00910737823460548 0.721037399657979 -3.96471835996519 |
| Mir7031 0.393774326892446 6.67118281898593 4.21183001468067 0.00550357754031352 0.721037399657979 -3.90790944101807 |
| Gm1401 0.367736455676756 4.76317061201306 3.75382866322948 0.00931578888724868 0.721037399657979 -3.96745224563801 |

**Table S2: TesaNPY_vs_FP_NPY_downregulated genes**

| logFC "AveExpr" "t" "P.Value" "adj.P.Val" "B" |
| --- |
| Lnx2 -0.357216861428104 9.09101410016986 -3.82513568028257 0.00856480597675748 0.721037399657979 -3.95737749072347 |
| Gm27342 -0.381317930037916 5.00467947928401 -3.91405032103725 0.0077210698523209 0.721037399657979 -3.94524870886357 |
| Ypel2 -0.399957417328779 9.17659074181802 -3.93360811232074 0.00754815864922318 0.721037399657979 -3.94264378300428 |
| Mir376a -0.403683897477025 5.5808412841423 -3.78644808895517 0.00896353986901868 0.721037399657979 -3.96280442698721 |
| Gm12158 -0.405217151961765 6.58888644629698 -3.85000725651916 0.00831889511803909 0.721037399657979 -3.95393694838052 |
| Gm5460 -0.407199823491197 6.09174141013655 -3.95655186467037 0.00735078956404666 0.721037399657979 -3.93961625744806 |
| Pde7b -0.426416483907681 9.06696766040633 -3.86929244374012 0.00813362043094901 0.721037399657979 -3.9512949187149 |
| Cttnbp2nl -0.427494122398521 10.5734935319708 -3.70398288141004 0.00988411278165222 0.721037399657979 -3.97468417077194 |
| Isca1 -0.447584577693888 8.28012759772709 -4.19008405942577 0.00563878767573157 0.721037399657979 -3.91047433040464 |
| Kitl -0.448142198852548 10.7610714988208 -4.42607395660336 0.00434936005226914 0.721037399657979 -3.88389981868725 |
| Ginm1 -0.454375409738571 10.7335238707554 -3.70358077384634 0.00988885108258877 0.721037399657979 -3.97474315524744 |
| Dtx2 -0.456749343529215 8.11009062766169 -4.1717417089748 0.00575573125823772 0.721037399657979 -3.91265682996484 |
| Tmem245 -0.461973161358123 11.5423015664926 -3.82967862222982 0.00851929203485436 0.721037399657979 -3.95674624839406 |
| Ric3 -0.467364930090329 6.86700867173651 -3.94978207771886 0.00740842046760355 0.721037399657979 -3.94050638954585 |
| Nuak1 -0.467828191519995 8.57515009898832 -4.11598432549341 0.00612822208333792 0.721037399657979 -3.91939990494654 |
| Dnajc4 -0.470630279578507 10.3703722508798 -4.05855423692821 0.00654031031843742 0.721037399657979 -3.92651972902903 |
| Kat2b -0.472244875453487 10.6570687165618 -4.29067750813065 0.00504293114807723 0.721037399657979 -3.89881137835793 |
| Trnp1 -0.473469865162048 5.93480597853947 -3.71539629330195 0.00975066496395955 0.721037399657979 -3.973014279693 |
| Inf2 -0.478426869967182 9.19502656045867 -4.39808379342814 0.00448346177145086 0.721037399657979 -3.88691083568776 |
| Lrrc8c -0.481131903733624 10.7336388885678 -3.89958961811523 0.00785175516389091 0.721037399657979 -3.9471891842109 |
| Pcolce2 -0.485582154480292 8.87764822275315 -4.70143480831917 0.00324559996285951 0.721037399657979 -3.85614290185224 |
| Gm10702 -0.487935492133778 6.61728659407807 -4.58262092419795 0.00367767484475636 0.721037399657979 -3.86771648575011 |
| Mpp7 -0.490686875214877 8.50075551577976 -4.46270787351945 0.00418060756608908 0.721037399657979 -3.88001383303579 |
| Adra1d -0.490769210690061 7.93178454803307 -3.90636532005329 0.00779021709268806 0.721037399657979 -3.94627842153171 |
| Gm19918 -0.491319653620579 5.50116809782827 -4.04191971586319 0.00666538791771285 0.721037399657979 -3.92861569567395 |
| Bag6 -0.495150293314103 9.81800208550798 -3.83422270698278 0.00847403549109829 0.721037399657979 -3.95611610503352 |
| Gga2 -0.499581942686368 7.98073818989781 -3.74847303587455 0.00937507852925988 0.721037399657979 -3.96822172093717 |
| Mmp28 -0.499687947570269 7.76221894668566 -4.00130143494515 0.00698217998432235 0.721037399657979 -3.93379840097617 |
| Olfr995 -0.500608236608285 4.92484668646483 -3.86576035868407 0.00816720723148191 0.721037399657979 -3.95177713514329 |
| Gm13294 -0.500977350236636 8.00377783746735 -4.81140693458936 0.00289614787516439 0.721037399657979 -3.84594299249367 |
| Tprgl -0.503600627256887 10.0165489052352 -4.29830928378968 0.00500068972270751 0.721037399657979 -3.89794727654865 |
| Plpp1 -0.504099952389468 10.6403111166205 -4.05343595545898 0.00657851403162834 0.721037399657979 -3.92716300909092 |
| Zdhhc8 -0.504648464585164 8.65035287080491 -3.89919366634137 0.00785536796995917 0.721037399657979 -3.94724249030784 |
| Ccdc85a -0.508396069766025 7.02085348246288 -3.7200648947663 0.00969665549122531 0.721037399657979 -3.9723336201127 |
| Cobll1 -0.51127791643878 9.51889336194099 -3.77970918273513 0.00903508176460293 0.721037399657979 -3.96375918761068 |
| Gm20335 -0.51177343189088 6.15879561842755 -3.75397321375866 0.00931419441385449 0.721037399657979 -3.96743150221412 |
| Lmtk2 -0.516627721629012 9.25946872634046 -4.38312920379163 0.0045570100337459 0.721037399657979 -3.88853463747737 |
| Ctdspl -0.521746581555291 9.38142567206675 -4.1076269004582 0.00618634440003529 0.721037399657979 -3.9204248918899 |
| Bcl9l -0.52479929680889 9.07884604722842 -3.73412592007925 0.00953598244116596 0.721037399657979 -3.9702919789137 |
| Phf8 -0.528865077750648 9.45240405077698 -4.19604493163051 0.00560135818303394 0.721037399657979 -3.90976882854648 |
| Hspb6 -0.529044142119045 9.17342325823497 -3.95784263936772 0.00733985808212425 0.721037399657979 -3.93944683881816 |
| Fstl3 -0.530522548263369 7.34404379276218 -3.77726390149343 0.00906119809022294 0.721037399657979 -3.96410633058099 |
| LOC100862103 -0.530718487697751 8.95580387466795 -3.94056661850877 0.00748768237759067 0.721037399657979 -3.94172235380905 |
| Nacc2 -0.532655077376239 8.46749917700611 -3.96292831042465 0.00729696392327882 0.721037399657979 -3.93878026066075 |
| Gm9795 -0.533221767629844 7.03910221155396 -4.82458713339004 0.00285719542698874 0.721037399657979 -3.84475231255291 |
| Atpaf2 -0.535021964966284 8.47895987648288 -4.39438664274574 0.00450151984644253 0.721037399657979 -3.88731129845658 |
| Ints7 -0.536363158979189 9.94590608959195 -3.95140480806848 0.00739456047828224 0.721037399657979 -3.94029278206938 |
| Slc39a13 -0.538111227620528 8.40931614834746 -4.08904233838062 0.00631781436451996 0.721037399657979 -3.92271768105428 |
| Fam168a -0.541929559722193 8.92033177737063 -4.29737222088353 0.0050058548714108 0.721037399657979 -3.89805321987574 |
| Extl3 -0.542534816082438 12.0533198034493 -3.78516630675662 0.00897709898780663 0.721037399657979 -3.96298581119147 |
| Zcchc24 -0.543324992420471 10.3328847511515 -4.2737066478218 0.0051383060935333 0.721037399657979 -3.90074323740048 |
| Olfr1034 -0.556699665066093 7.64118809130118 -3.93189438026078 0.00756313607137583 0.721037399657979 -3.94287114383576 |
| Fam124a -0.559636739791116 7.78112320985772 -4.20285630069751 0.00555892848842377 0.721037399657979 -3.90896491520193 |
| Map4 -0.559968714353485 11.6975222327654 -4.30744017887536 0.00495067146114938 0.721037399657979 -3.89691721107242 |
| Sh3glb2 -0.560802022817101 8.204612680071 -4.24497543413871 0.00530441999453553 0.721037399657979 -3.90404663456965 |
| Grina -0.561431110850331 14.4273037920619 -3.90617126215521 0.00779197207016002 0.721037399657979 -3.94630446834099 |
| Tspan7 -0.567658372617548 11.506486956529 -3.90200461600819 0.00782975987327602 0.721037399657979 -3.9468642590487 |
| Pnpla2 -0.567892241666117 16.7527570090264 -3.92372502378684 0.00763499007453406 0.721037399657979 -3.94395732731691 |
| Tnpo2 -0.568264161385089 9.61256408799209 -4.75977232537947 0.00305463540280238 0.721037399657979 -3.85067249732017 |
| Tmbim1 -0.573313278990009 10.1847186009948 -3.81269891198943 0.00869079398141287 0.721037399657979 -3.95911203321451 |
| Gpr108 -0.573747473766597 10.3279769311674 -4.21820025933214 0.00546465981033958 0.721037399657979 -3.9071626931301 |
| Cdk16 -0.576406697936227 10.8874066954993 -5.1399613318743 0.0020809469255479 0.721037399657979 -3.81815238351581 |
| Ephx3 -0.577779579402973 6.37198583909534 -4.62064073140623 0.00353272844295756 0.721037399657979 -3.86394849727281 |
| Hey1 -0.582891448336231 7.4521780494861 -4.31566869919331 0.00490607683147462 0.721037399657979 -3.89599245077955 |
| Slc25a28 -0.583889876266303 8.27682611490167 -5.03197305488875 0.00231609378784462 0.721037399657979 -3.8268664154436 |
| LOC100862120 -0.58565679260217 6.6923406249978 -4.04391924422036 0.00665021279934235 0.721037399657979 -3.92836294334843 |
| Fam132a -0.592952907436229 8.01325467575371 -3.85356454753387 0.00828436967363481 0.721037399657979 -3.95344792317039 |
| Mef2d -0.594882935506356 10.4113227263551 -4.86294310722663 0.00274716638068084 0.721037399657979 -3.84132499175499 |
| Sorbs1 -0.596551516466309 12.5204980632336 -5.01765673068518 0.002349471783492 0.721037399657979 -3.82805150920824 |
| Gm16400 -0.599151806949709 5.58894951994786 -4.42484605239112 0.00435514753648702 0.721037399657979 -3.88403114310941 |
| Zfp423 -0.600488811735871 8.76867227473366 -3.87371032395286 0.00809182671563607 0.721037399657979 -3.95069282110694 |
| Kctd17 -0.601969318128983 8.35656807839933 -3.80026456054098 0.00881881996884227 0.721037399657979 -3.96085572695556 |
| Gm23482 -0.604494354202041 6.63075349191508 -3.9778271008662 0.0071729032184143 0.721037399657979 -3.93683602439722 |
| Gm5144 -0.609966339813714 7.31758605774862 -3.73681156372946 0.00950563114847739 0.721037399657979 -3.96990345556737 |
| Adcy6 -0.61013731697424 10.1273673266494 -4.64753558603183 0.00343408293269618 0.721037399657979 -3.8613200366024 |
| Ampd3 -0.612685870679111 10.2768347768257 -4.27325788588924 0.00514085541134791 0.721037399657979 -3.90079451619475 |
| LOC100504971 -0.618305169529362 8.21985405439261 -5.47742083746765 0.00150365905659033 0.721037399657979 -3.7933014363673 |
| Eng -0.620589406814033 11.8322182160555 -4.02346027182809 0.00680732400775889 0.721037399657979 -3.93095958475708 |
| Gm15378 -0.628431329945274 10.3817725040861 -3.69934100215825 0.00993896465925838 0.721037399657979 -3.97536571241556 |
| Vmn1r-ps149 -0.629963456920098 6.2991451974249 -4.57652994320023 0.00370151324952889 0.721037399657979 -3.86832589423211 |
| Amotl1 -0.636214682475049 9.85115152723754 -4.06004436219981 0.00652923440194113 0.721037399657979 -3.92633271722412 |
| 4930402H24Rik -0.639617586168043 10.747345808229 -4.54977327838246 0.00380831399298558 0.721037399657979 -3.87102196888229 |
| Fbxw8 -0.64215382428231 10.0788889063471 -4.68934202742125 0.00328685023028335 0.721037399657979 -3.8572940498852 |
| Sav1 -0.643609795124039 11.5024025257441 -3.70277567250326 0.00989834569687565 0.721037399657979 -3.97486128540563 |
| Dll4 -0.64896769826052 9.14983309638097 -3.81063634267903 0.00871188686599501 0.721037399657979 -3.95940061370191 |
| S1pr1 -0.651179999129188 8.22031033837614 -4.67847934955644 0.00332440934032064 0.721037399657979 -3.85833319946157 |
| Eif4ebp1 -0.656752898439494 11.1063276324159 -3.90492696458348 0.007803235438846 0.721037399657979 -3.94647153301022 |
| Fzd5 -0.658699247559025 7.35853952950478 -4.67289558739108 0.00334390417846587 0.721037399657979 -3.858869241843 |
| Klf6 -0.659337035501183 13.149490780986 -4.1635983648629 0.00580851835520925 0.721037399657979 -3.91363141309742 |
| Tcta -0.663722210722401 8.2240319016508 -4.20347829634405 0.00555507189653959 0.721037399657979 -3.90889162333154 |
| Cdc42ep2 -0.665212740040019 6.46252371248106 -3.89998325403847 0.00784816532238404 0.721037399657979 -3.94713619907771 |
| Anxa6 -0.681819629738184 13.7388368198446 -4.64865389958768 0.00343004893797632 0.721037399657979 -3.86121139923167 |
| Sort1 -0.682370898426285 12.8299311428745 -4.43829628155473 0.00429222149883825 0.721037399657979 -3.88259644941139 |
| Natd1 -0.684634204391957 7.21470835109637 -5.38410005889116 0.00164267514167351 0.721037399657979 -3.79983319423372 |
| Wipi1 -0.686831674018452 10.1697802504975 -3.7705133689221 0.00913373194820273 0.721037399657979 -3.96506660058794 |
| Prelp -0.692393087840255 17.9096603035181 -3.90260580029109 0.00782429508578495 0.721037399657979 -3.9467834264402 |
| Zfand3 -0.693370146755412 11.8223422897778 -4.55313940112727 0.00379468892504727 0.721037399657979 -3.87068107495816 |
| Ubap2 -0.6966726600737 12.5933613786558 -4.66556263251975 0.00336970223906495 0.721037399657979 -3.85957516026776 |
| Ppp2r1b -0.702318412892253 10.8933446753104 -4.54005653443117 0.00384795456892453 0.721037399657979 -3.87200878669961 |
| Acer2 -0.707318700058082 10.269701527339 -4.23094916273166 0.0053876954987172 0.721037399657979 -3.90567444387656 |
| Pxdn -0.711786351221462 11.0819610684037 -5.51249518020076 0.00145490894107616 0.721037399657979 -3.79090981766335 |
| Cav1 -0.718155776894762 15.4999288728696 -4.22438412786465 0.00542717541352441 0.721037399657979 -3.9064397802272 |
| Plxna2 -0.719872052769701 10.005535088337 -4.7626473897052 0.00304555740731064 0.721037399657979 -3.85040640760499 |
| Gm16437 -0.720297607721216 11.2205966781755 -3.88745637305169 0.00796330848174763 0.721037399657979 -3.94882687234486 |
| Myo1c -0.72121157148582 11.1223471735379 -4.19885530696591 0.00558380786340671 0.721037399657979 -3.90943684302328 |
| Adcy5 -0.721516969847159 9.91752152253674 -4.05667811887126 0.00655428513159762 0.721037399657979 -3.9267553571478 |
| Rras -0.724338554562471 14.9320232853693 -4.54076654638325 0.00384504231005808 0.721037399657979 -3.87193653863888 |
| 1700037H04Rik -0.731286354805796 9.58095486292618 -4.66619907676259 0.00336745429033223 0.721037399657979 -3.85951380390291 |
| Rassf3 -0.73212261974629 9.56885053355169 -4.72531495215333 0.00316584578013822 0.721037399657979 -3.85388711224355 |
| Gm3470 -0.742880804957815 10.6559627263417 -5.74399887460611 0.00117480564196334 0.721037399657979 -3.77594325048817 |
| Gm4876 -0.745467728917773 7.58967285891181 -4.35227472718618 0.00471307838008613 0.721037399657979 -3.8919184504175 |
| Gm2399 -0.745660811478779 15.8067286990591 -4.52256139197038 0.00392050501161573 0.721037399657979 -3.8737960438039 |
| Rgcc -0.747281164656285 12.5151155787297 -5.15392419047789 0.00205256783678439 0.721037399657979 -3.81705405190634 |
| Il34 -0.748887379681454 7.15128481984772 -4.29644744264655 0.0050109581750581 0.721037399657979 -3.89815781678273 |
| 5430416O09Rik -0.752791723554656 6.54169476894573 -4.2344147963668 0.00536698420311494 0.721037399657979 -3.90527131340083 |
| Lnx1 -0.752987389019667 7.18613351713827 -5.8544707063895 0.00106318342634261 0.721037399657979 -3.7692732423542 |
| Gnpat -0.759054873561451 12.3069943936169 -3.8018949014549 0.00880191496566215 0.721037399657979 -3.96062655881613 |
| Cxx1b -0.762420735129218 12.2996558303612 -5.37195016853999 0.00166182723968978 0.721037399657979 -3.80070202960544 |
| LOC100861951 -0.764822872230647 5.45078063984765 -4.49635525669594 0.00403207795851177 0.721037399657979 -3.8764986122432 |
| Nid1 -0.77042621095443 14.6394112025738 -3.9217970031916 0.00765205880336549 0.721037399657979 -3.94421424241339 |
| Pea15a -0.778705689534541 10.437981627441 -3.78656656387942 0.00896228775069333 0.721037399657979 -3.96278766682165 |
| Tpcn1 -0.787452915213304 12.5778547127819 -3.80276878271523 0.00879286851615244 0.721037399657979 -3.96050378954648 |
| C130074G19Rik -0.791192954877861 8.87510365996526 -4.39673319250742 0.00449004908809532 0.721037399657979 -3.8870570535736 |
| Sdpr -0.79329107857088 13.0061562789764 -3.97426108601214 0.00720238149091893 0.721037399657979 -3.93730021881994 |
| Itih5 -0.794723989853346 14.5543780637781 -4.68648113676212 0.00329669547515491 0.721037399657979 -3.85756726026432 |
| Entpd2 -0.800062687758034 8.72294148280276 -3.9191476589659 0.00767558277939575 0.721037399657979 -3.94456763125059 |
| Ptrf -0.803955143507883 11.7224750515468 -4.08339838903535 0.00635835657914751 0.721037399657979 -3.92341768563865 |
| Htra1 -0.805477539159641 9.31887767290375 -5.04511999541955 0.00228591467424365 0.721037399657979 -3.82578437262625 |
| LOC100861758 -0.810816644842548 7.81032906494647 -5.19692072757002 0.00196789845248482 0.721037399657979 -3.81371165197539 |
| S100a11 -0.814325937757464 18.5085239603581 -4.77528154831628 0.0030060246285415 0.721037399657979 -3.84924096641014 |
| Gm25602 -0.814781108412037 5.1486209491056 -4.39816908735254 0.0044830461301499 0.721037399657979 -3.88690160453327 |
| Slc9a3r2 -0.816680182972386 9.1354144245808 -6.01920448252008 0.000918474304083981 0.721037399657979 -3.75984912264133 |
| Kirrel -0.836847514011814 10.2234771761248 -4.28192224545646 0.00509188475372298 0.721037399657979 -3.89980623691683 |
| Lipe -0.842817908001119 13.3336039037692 -3.953136284093 0.00737980354233266 0.721037399657979 -3.94006502768622 |
| LOC100861980 -0.84706628343497 11.647259371313 -4.01385827608829 0.00688248633532403 0.721037399657979 -3.93218632758888 |
| LOC100861791 -0.864830182213956 7.95690153917246 -5.75860143674605 0.00115931074899724 0.721037399657979 -3.77504478331675 |
| Ltbp3 -0.865230292846669 9.82571234945051 -4.09488471504033 0.00627615089658811 0.721037399657979 -3.9219948887604 |
| Prkar2b -0.86862897658014 14.8517578637962 -4.0911735223446 0.0063025806377106 0.721037399657979 -3.92245380608628 |
| Sema7a -0.868754371024417 8.16798071929463 -3.93030735343605 0.00757703567195259 0.721037399657979 -3.94308184732884 |
| Gm19274 -0.869153883440963 7.55414817977597 -6.40203485228155 0.000661197251281996 0.721037399657979 -3.74012559764107 |
| Cd99 -0.869450404287004 8.89754896618098 -4.32819813930674 0.00483903647039939 0.721037399657979 -3.894590688986 |
| Lims2 -0.872978501007475 12.1786697333668 -7.8937396032174 0.000209768427810163 0.721037399657979 -3.68483337421155 |
| LOC100862171 -0.874902555831403 6.01333704268091 -4.09937770683645 0.00624431912924594 0.721037399657979 -3.92144029445779 |
| Lgals12 -0.888300835442576 12.2675586030607 -3.80317354473299 0.00878868190661214 0.721037399657979 -3.96044694154686 |
| Arhgef37 -0.896996129985248 8.13701943982569 -4.29740672817529 0.00500566455897176 0.721037399657979 -3.89804931775021 |
| Prrx1 -0.898684654889495 9.62984898519348 -4.19466284098011 0.00561001176104926 0.721037399657979 -3.90993224270321 |
| Tenm4 -0.915744685066199 9.52978350649096 -5.01468632941297 0.00235646522496721 0.721037399657979 -3.82829828994234 |
| Notch3 -0.921375570600417 9.35259171369443 -4.34142973197044 0.00476935325221202 0.721037399657979 -3.89311865825509 |
| Gm3211 -0.927871089912941 6.44552977889909 -4.903155748393 0.00263692603664846 0.721037399657979 -3.83779113712801 |
| Large -0.945185102383253 10.4586987220854 -5.16728541583219 0.00202582193027941 0.721037399657979 -3.81600899921206 |
| Npr3 -0.945651357690177 15.3835413628915 -3.88776390664176 0.00796045931038173 0.721037399657979 -3.94878525505279 |
| LOC100861999 -0.959798946407914 8.05625054826222 -5.01002647328641 0.0023674838386415 0.721037399657979 -3.82868605125226 |
| Irs3 -0.966977151380557 8.30068055344619 -4.77953374180986 0.00299284996787053 0.721037399657979 -3.84885013214741 |
| Sgsh -0.970718381165403 8.33947654683645 -3.70005095089676 0.00993055356738281 0.721037399657979 -3.97526138481783 |
| Fgfrl1 -0.984179014700465 9.7711698809697 -6.14890045459236 0.000820264007677199 0.721037399657979 -3.75284182360167 |
| Plin1 -0.988710545782584 13.1022807187599 -4.38060263930155 0.00456956931516687 0.721037399657979 -3.88881002156124 |
| Cd151 -0.99294794157741 13.4295204767098 -3.87776870116786 0.00805364447363448 0.721037399657979 -3.95014074714718 |
| Cnnm2 -1.01141384511321 9.88760931590646 -3.78316363804663 0.00899832950919724 0.721037399657979 -3.9632694117933 |
| Pde2a -1.01417924749842 12.5424082753337 -5.79817497543415 0.00111847705890382 0.721037399657979 -3.77263586306487 |
| Fam101b -1.01590948033897 8.37718186007941 -4.68840987567206 0.00329005441599396 0.721037399657979 -3.85738303206097 |
| Gm24157 -1.02061677163079 7.45542208913526 -4.34329389463547 0.0047596266170555 0.721037399657979 -3.89291194902399 |
| Fry -1.03501731852091 11.0129506473309 -4.89393616393864 0.00266175184973448 0.721037399657979 -3.83859603764953 |
| Tln2 -1.04240294907302 9.54863960981855 -4.20714835588992 0.00553237715697026 0.721037399657979 -3.9084595734516 |
| Aoc3 -1.04591804191377 14.0543144501125 -4.72752496339186 0.00315857738494126 0.721037399657979 -3.85367951089436 |
| Pparg -1.05574460428889 12.9023157402666 -4.52154806851937 0.00392475410073977 0.721037399657979 -3.87389997683254 |
| E030003E18Rik -1.06070895018577 8.72917122619974 -4.22832870624976 0.00540341532314819 0.721037399657979 -3.90597966715219 |
| Pam -1.07296061840325 12.3099368456382 -3.86105065085257 0.00821223243880871 0.721037399657979 -3.95242129039824 |
| Ehd2 -1.0908437520303 13.6473193190273 -4.2218290672606 0.00544262823871754 0.721037399657979 -3.90673823756396 |
| Prkcdbp -1.12087189084982 10.1926575324261 -6.2258073032109 0.000767717039987822 0.721037399657979 -3.74884842989307 |
| Fads3 -1.1266534123256 15.5859049005205 -3.75939091649398 0.00925465197975035 0.721037399657979 -3.96665499301697 |
| Hspg2 -1.13748522210359 13.9307027484416 -4.91518043501419 0.0026049395171404 0.721037399657979 -3.83674604806047 |
| Aqp7 -1.16380760669685 13.757381689022 -4.30063133621197 0.00498791617278759 0.721037399657979 -3.89768493434613 |
| Igfbp5 -1.18013345426989 13.0263876166008 -4.63692554746461 0.00347262291543126 0.721037399657979 -3.86235333836937 |
| Gm19383 -1.20206647358769 8.56186800178402 -3.94003983866 0.00749224167184021 0.721037399657979 -3.94179201030836 |
| Fzd4 -1.2329222002979 14.2952028186841 -5.60237748381496 0.00133798212062279 0.721037399657979 -3.7849334486117 |
| Htra3 -1.23439689147066 13.5157859132103 -3.84757097841895 0.00834263285667838 0.721037399657979 -3.95427230740459 |
| Rhob -1.23535953091679 10.8838705571813 -7.26011778003626 0.000333600081164381 0.721037399657979 -3.70486246603873 |
| Tsc22d2 -1.24502070321215 10.435378194055 -4.07638153730207 0.00640916680814601 0.721037399657979 -3.92429038754027 |
| Hspb7 -1.28877462350513 11.1673134813112 -3.96367138915542 0.00729071999643327 0.721037399657979 -3.9386829900176 |
| Folh1 -1.32711113555784 8.85000794157508 -4.9282173871249 0.00257075528383487 0.721037399657979 -3.83561896683388 |
| Fhl5 -1.40815318111451 7.98949699147563 -4.3297353011567 0.00483088273592155 0.721037399657979 -3.8944192418393 |
| Tmem120b -1.46590667014799 12.2254356409351 -3.77560226222741 0.00907899272270923 0.721037399657979 -3.96434243676922 |
| 2610016A17Rik -1.54728234359936 8.52132962815631 -4.02477933452946 0.00679707044316835 0.721037399657979 -3.93079146597569 |
| Cryab -1.84688464626126 12.2739615467012 -4.19955814647817 0.00557942838209879 0.721037399657979 -3.90935388141387 |
| Ehd3 -1.84872916191621 10.904973105319 -5.71753817027048 0.00120348874022422 0.721037399657979 -3.77758465502481 |

**Table S3**: **KEGG pathway analysis identified pathways** of differentially expressed genes was related to lipid metabolism (fatty acid degradation, increased arachidonic and linoleic acid metabolism), PPAR signaling pathway, drug metabolism as well as insulin signaling pathway which was down regulated between animals treated with Tesa-NPY and [F7, P34]-NPY of genes.

KEGG Pathways were plotted using Pathview package (Weijun Luo and Cory Brouwer. Pathview: A R/Bioconductor package for pathway-based data integration and visualization. Bioinformatics, 29(14):1830–1831, 2013. doi: 10.1093/bioinformatics/btt285. URL http: //[bioinformatics.oxfordjournals.org/content/29/14/1830.full](http://bioinformatics.oxfordjournals.org/content/29/14/1830.full).)

| **Pathway** | **p.geomean** | **stat.mean** | **p.val** | **q.val** | **set.size** |
| --- | --- | --- | --- | --- | --- |
| mmu00982  Drug metabolism - cytochrome P450 | 1.27912591509344e-10 | 7.13 | 1.27912591509345e-10 | 2.08497524160232e-08 | 77 |
| mmu00830  Retinol metabolism | 1.27471164157153e-09 | 6.78 | 1.27471164157153e-09 | 1.0388899878808e-07 | 68 |
| mmu00980  Metabolism of xenobiotics by cytochrome P450 | 1.86482113496999e-08 | 6.12 | 1.86482113496998e-08 | 1.01321948333369e-06 | 67 |
| mmu00983  Drug metabolism - other enzymes | 3.43310887480913e-06 | 4.96 | 3.43310887480912e-06 | 0.0001398992 | 49 |
| mmu04610  Complement and coagulation cascades | 4.35819809895608e-06 | 4.73 | 4.35819809895609e-06 | 0.0001420773 | 76 |
| mmu00591  Linoleic acid metabolism | 1.59835701379735e-05 | 4.57 | 1.59835701379735e-05 | 0.0004342203 | 44 |
| mmu00071  Fatty acid metabolism | 5.24291148098703e-05 | 4.21 | 5.24291148098703e-05 | 0.0010590411 | 47 |
| mmu00140  Steroid hormone biosynthesis | 5.37917464355258e-05 | 4.17 | 5.37917464355258e-05 | 0.0010590411 | 47 |
| mmu00590  Arachidonic acid metabolism | 5.84746619855609e-05 | 4.02 | 5.8474661985561e-05 | 0.0010590411 | 88 |
| mmu03320  PPAR signaling pathway | 9.51381374590019e-05 | 3.90 | 9.51381374590018e-05 | 0.0015507516 | 79 |
